# Prenylation of dimeric *cyclo*-l-Trp-l-Trp by the promiscuous *cyclo*-l-Trp-l-Ala prenyltransferase EchPT1

**DOI:** 10.1007/s00253-023-12773-0

**Published:** 2023-09-15

**Authors:** Wen Li, Xiulan Xie, Jing Liu, Huili Yu, Shu-Ming Li

**Affiliations:** 1 Institut für Pharmazeutische Biologie und Biotechnologie, Fachbereich Pharmazie, Philipps-Universität Marburg, Robert-Koch-Straße 4, 35037 Marburg, Germany; 2 Fachbereich Chemie, Philipps-Universität Marburg, Hans-Meerwein-Straße 4, 35032 Marburg, Germany

**Keywords:** Dimeric cyclodipeptides, Prenylation, Dimethylallyl tryptophan synthase, EchPT1, Biocatalyst

## Abstract

**Abstract:**

Prenyltransferases (PTs) from the dimethylallyl tryptophan synthase (DMATS) superfamily are known as efficient biocatalysts and mainly catalyze regioselective Friedel-Crafts alkylation of tryptophan and tryptophan-containing cyclodipeptides (CDPs). They can also use other unnatural aromatic compounds as substrates and play therefore a pivotal role in increasing structural diversity and biological activities of a broad range of natural and unnatural products. In recent years, several prenylated dimeric CDPs have been identified with wide range of bioactivities. In this study, we demonstrate the production of prenylated dimeric CDPs by chemoenzymatic synthesis with a known promiscuous enzyme EchPT1, which uses *cyclo*-l-Trp-l-Ala as natural substrate for reverse *C2*-prenylation. High product yields were achieved with EchPT1 for C3-N1′ and C3-C3′ linked dimers of *cyclo*-l-Trp-l-Trp. Isolation and structural elucidation confirmed the product structures to be reversely C19/C19′-mono- and diprenylated *cyclo*-l-Trp-l-Trp dimers. Our study provides an additional example for increasing structural diversity by prenylation of complex substrates with known biosynthetic enzymes.

**Key points:**

*• Chemoenzymatic synthesis of prenylated* *cyclo*-l-Trp-l-Trp* dimers*

*• Same prenylation pattern and position for cyclodipeptides and their dimers*.

*• Indole prenyltransferases such as EchPT1 can be widely used as biocatalysts*.

**Graphical Abstract:**

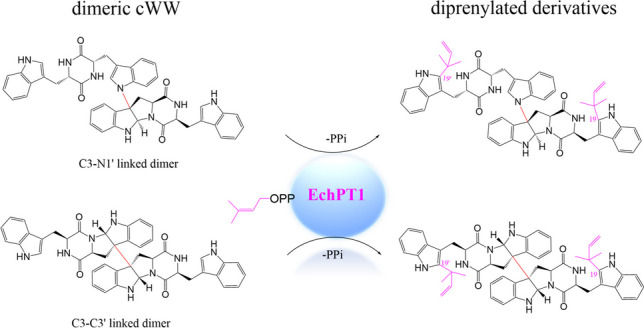

**Supplementary Information:**

The online version contains supplementary material available at 10.1007/s00253-023-12773-0.

## **Introduction**

**Fig. 1:**
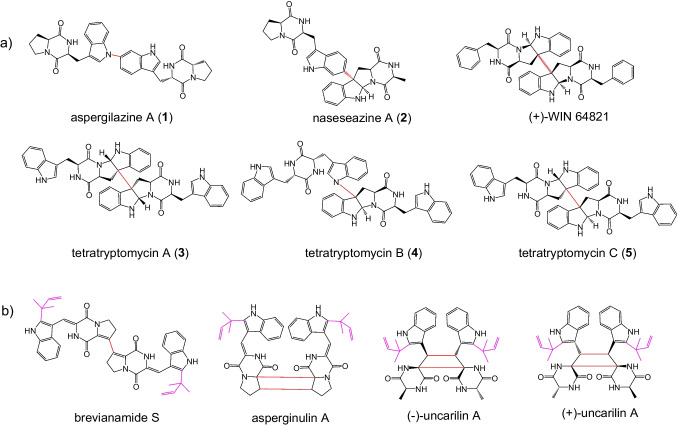
Examples of known dimeric tryptophan-containing cyclodipeptides (**a**) and prenylated ones (**b**)

Indole alkaloids derived from tryptophan-containing cyclodipeptides (CDPs) with a 2,5-diketopiperazine skeleton were isolated from various microorganisms and plants and are well-known for their structural diversity and pharmaceutical utility (Goetz et al. 2011; Li 2010; Lindel et al. 2012; Vinokurova et al. 2003; Xu et al. 2014). Among them, tryptophan-based dimeric diketopiperazine alkaloids have been identified in recent years and their biosynthetic pathways have been elucidated (Gerken and Walsh 2013; Harken and Li 2021; Kim and Movassaghi 2011; Kim and Movassaghi 2015). Typically, these natural products are biosynthesized from tryptophan and another amino acid such as tryptophan, proline, alanine, or valine. The two amino acids are mainly condensed by either a nonribosomal peptide synthetase in fungi or a cyclodipeptide synthase in bacteria, resulting in the formation of the two peptide bonds. The CDP core is then modified by cytochrome P450 to generate the homo- or heterodimer with unique bond connection (Gomes et al. 2019; Harken and Li 2021; Malit et al. 2021). Examples are the C6-N1′ linked aspergilazine A (**1**), the C3-C6′ linked naseseazine A (**2**), and tetratryptomycins A–C (**3**–**5**) with the symmetrical C3-C3′ and unsymmetrical C3-N1′ linkage (Fig. 1a) (Liu et al. 2020; Yu and Li 2019). The C3-C3′ linked (+)-WIN 64821 was isolated from *Aspergillus versicolor* and exhibits potential analgesic and anti-inflammatory activities by inhibition of substance P receptor (Fig. 1a) (Movassaghi et al. 2008; Tadano et al. 2013).

Recently, several tryptophan-containing dimeric CDPs carrying prenyl moieties were also isolated from different sources (Cai et al. 2019; Geng et al. 2017; Song et al. 2012). Interestingly, all these complex structures are *C2*-prenylated *cyclo*-l-Trp-l-Pro (cWP) or *cyclo*-l-Trp-l-Ala (cWA) dimers with different connections and symmetries. The cWP dimer brevianamide S exhibits selective antibacterial activity against Bacille Calmette-Guérin (BCG), which serves as a valuable lead for next-generation antitubercular drugs (Song et al. 2012). Another cWP dimer asperginulin A with an unprecedented 6/5/4/5/6 pentacyclic skeleton showed obvious toxicity in inhibiting settlement of the larvae of *Balanus reticulatus* (Cai et al. 2019). (+)/(−)-Uncarilin A, a pair of dimeric isoechinulin-type enantiomers with a symmetric four-membered core, was isolated from *Uncaria rhynchophyl* as a new type of melatonin receptors (Fig. 1b) (Geng et al. 2017). Attachment of prenyl moieties onto the indole nucleus usually increases the spectrum of the biological activities. However, in sharp contrast to the structural diversity of dimeric CDPs and CDP prenyltransferases (PTs), there are rare examples of prenylating enzymes toward CDP dimers.

**Fig. 2:**
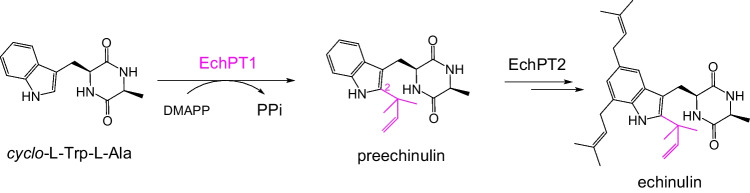
Prenylation of *cyclo*-l-Trp-l-Ala by EchPT1 in the biosynthesis of echinulin

The members of the dimethylallyl tryptophan synthase (DMATS) superfamily as important biocatalysts usually catalyze metal ion-independent Friedel-Crafts prenylations. They use predominantly tryptophan and other indole derivatives as prenyl acceptors but can also accept a broad spectrum of aromatic compounds for prenylation. They were therefore already used for structural modification of diverse small molecules (Fan et al. 2015). By using the fungal PT CdpC3PT and its mutants, Xu successfully obtained prenylated biflavonoids (Xu et al. 2021). In this study, we intended to get prenylated dimeric CDPs by these soluble PTs. Our previous studies demonstrated that the *cyclo*-l-Trp-l-Ala (cWA) *C2-*prenyltransferase EchPT1 is involved in the biosynthesis of echinulin (Fig. 2) (Wohlgemuth et al. 2017) and shows a high flexibility toward different substrates (Li et al. 2023; Wohlgemuth et al. 2018). Accordingly, we selected this enzyme and four additional DMATS PTs for prenylation of dimeric CDPs. One mono-and three diprenylated *cyclo*-l-Trp-l-Trp (cWW) dimers were obtained in high conversion yields.

## Materials and methods

### Chemicals

Dimethylallyl diphosphate (DMAPP) was chemically synthesized according to the method published previously (Woodside et al. 1988). Aspergilazine A (**1**), naseseazine A (**2**), and tetratryptomycins A–C (**3**–**5**) were isolated as described before (Liu et al. 2020; Yu and Li 2019).

### Strains, plasmids, and culture conditions

*Escherichia coli* strain BL21 (DE3) pLysS (Invitrogen, Karlsruhe, Germany) and M15 (pREP4) (Qiagen, Hilden, Germany) were used for gene expression and cultivated at 37 °C in Terrific broth (TB) medium. The plasmids pVW90, pALF49, pPM37, pJW12, and pLW40 were used for overproduction of the proteins EchPT1, FgaPT2_R244L, FgaPT2_Y398F, 6-DMATS_Sa_, and 7-DMATS, respectively (Fan and Li 2016; Kremer et al. 2007; Mai et al. 2016; Winkelblech and Li 2014; Wohlgemuth et al. 2017). To select the recombinant strains, ampicillin (50 μg/mL) and kanamycin (25 μg/mL) were added to the medium.

*Escherichia coli* ATCC 35218, *Enterococcus faecalis* DSM2570, *Klebsiella pneumoniae* DSM26371, *Bacillus subtilis* NCIB 3610, *Bacillus circulans* NRRL B-380, *Staphylococcus aureus* ATCC 29213, *Staphylococcus delphini* DSM20771, and *Pseudomonas aeruginosa* ATCC 27853 were used to evaluate the antibacterial activity.

### Protein purification and enzyme assays

Recombinant EchPT1, FgaPT2_R244L, FgaPT2_Y398F, 6-DMATS_Sa_, and 7-DMATS were purified by Ni-NTA affinity chromatography (Qiagen, Hilden) as described previously (Fan and Li 2016; Kremer et al. 2007; Mai et al. 2016; Winkelblech and Li 2014; Wohlgemuth et al. 2017). The purity of the five recombinant proteins was proven on 12% (w/v) SDS-PAGE (Li et al. 2023).

For enzyme reaction, standard assays (50 μL) contained Tris-HCl (50 mM, pH 7.5), CaCl_2_ (5 mM), dimeric CDP substrate (1 mM), DMAPP (1 mM), glycerol (0.5–5%, v/v), DMSO (2.5%, v/v), and the purified protein (7 μg). The reaction mixtures were incubated at 37 °C for 1 or 16 h and subsequently terminated with 50 μL MeOH. After centrifugation at 17,000 *×* g for 20 min, the enzyme reaction mixtures were analyzed on LCMS as described below.

Enzyme assays for product isolation were scaled up to a volume of 25 mL, containing Tris-HCl (50 mM, pH 7.5), CaCl_2_ (5 mM), DMAPP (1.5 mM), the respective dimeric CDP substrate (1 mM), and 5 mg purified EchPT1. The reaction mixtures were incubated at 37 °C for 16 h and extracted three times with two volumes of ethyl acetate each. The resulting organic phases were evaporated and dissolved in 1 ml MeOH for isolation.

The linearity of the EchPT1 reactions toward **3**–**5** was determined up to 360 min with 7 μg protein. To determine the kinetic parameters of EchPT1 toward the three cWW dimers, the enzyme assays (50 μL) contained Tris-HCl (50 mM, pH 7.5), CaCl_2_ (5 mM), DMAPP (1 mM), 7 μg EchPT1, and the cWW dimers at final concentrations of 0.01, 0.02, 0.05, 0.1, 0.2, 0.5, 1.0, and 2.0 mM. The reaction mixtures containing **3**, **4**, and **5** were incubated at 37 °C for 30, 30, and 20 min, respectively. For determination of the kinetic parameters of EchPT1 toward DMAPP in the presence of **3**, **4**, and **5**, the reaction mixtures contained Tris-HCl (50 mM, pH 7.5), CaCl_2_ (5 mM), the respective dimeric CDP substrate (1 mM), 7 μg EchPT1, and DMAPP at final concentrations from 0.01 to 2.0 mM, which were incubated at 37 °C for 30, 30, and 20 min, respectively. The reactions were terminated with 50 μL MeOH and analyzed on HPLC as described below. All the assays were performed as duplicates. The conversion yields were calculated by comparing with the isolated products as standard or by the ratio of the peak areas in HPLC chromatograms. The data were analyzed by using Prism 8.01 (GraphPad Software).

### LCMS and HPLC analysis of the enzyme products

LCMS analysis was performed as described previously (Zhou and Li 2021). The enzyme products were eluted at a flow rate of 0.25 mL/min with a linear gradient from 5 to 100% CH_3_CN in H_2_O in 10 min, followed by washing for 5 min and equilibration for 5 min. LCMS data were evaluated with DataAnalysis 4.2 software (Bruker Daltonik, Bremen, Germany).

For isolation of the target substances, semi-preparative HPLC was performed with an Agilent Eclipse XDB-C_18_ (250 × 9.4 mm, 5 μm) column. H_2_O (A) and CH_3_CN (B) were used as solvents at a flow rate of 2 mL/min. Compounds **3a2** and **5a2** were isolated with 80% B, **4a1** and **4a2** with 75% B. To determine the enzyme activities, an Agilent HPLC 1260 series equipped with a Multospher 120 RP-C_18_ (250 × 2 mm, 5 μm) column was used. H_2_O (A) and CH_3_CN (B) were used as mobile phase at a flow rate of 0.5 mL/min. The substances were eluted using a linear gradient from 5 to 100% B in A within 20 min.

### Structural elucidation of the enzyme products by NMR analysis

NMR spectra were recorded on a 500 MHz Bruker AVIII spectrometer and processed with MestReNova 6.1.0 (Metrelab). All the samples were dissolved in DMSO-*d*_6_ for measurement. Chemical shifts were referred to those of the solvent signals at δ_H_ 2.50 ppm and δ_C_ 39.5 ppm. The NMR data are provided in Tables S1–S4 and spectra in Figs. S7–S26.

### Antibacterial assays of the prenylated CDP dimers

The antibacterial activities of compounds **3a2**, **4a1**, **4a2**, and **5a2** were evaluated by using agar disk-diffusion method as reported previously (Balouiri et al. 2016). The eight bacteria strains were spread onto LB agar medium. Filter paper disks of about 5 mm in diameter were placed on the agar surface and 5 μL of 2 mM DMSO solution of the test compounds were dropped onto the paper disks. The inhibition growth zones around the disks were observed after incubation at 37 °C for 16 h. Kanamycin was used as positive control and DMSO (5 μL) was used as a negative control. All assays were performed in duplicates.

## Results

### Acceptance of five dimeric CDPs by DMATS PTs with different activities

The five PTs EchPT1, FgaPT2_R244L, FgaPT2_Y398F, 6-DMATS_Sa_, and 7-DMATS are responsible for the prenylation at C2, C4, C5, C6, and C7 of the indole ring of tryptophan and/or tryptophan-containing CDPs, respectively. To prove the acceptance of the tryptophan-containing dimeric CDPs by these enzymes, the recombinant proteins were incubated with cWP dimer apergilazine A (**1**) and cWA-cWP dimer naseseazine A (**2**) in the presence of DMAPP. LCMS analysis showed that formation of monoprenylated **1** with [M + H]^+^ ions at *m/z* 635.789 ± 0.005 and **2** at 607.735 ± 0.005 was only observed in the extracted ion chromatograms (EICs) of the reaction mixtures with EchPT1, FgaPT2_Y398F, and 7-DMATS (Figs. S1 and S2). Obviously, cWP-containing dimers are poor substrates of the tested PTs.

**Fig. 3:**
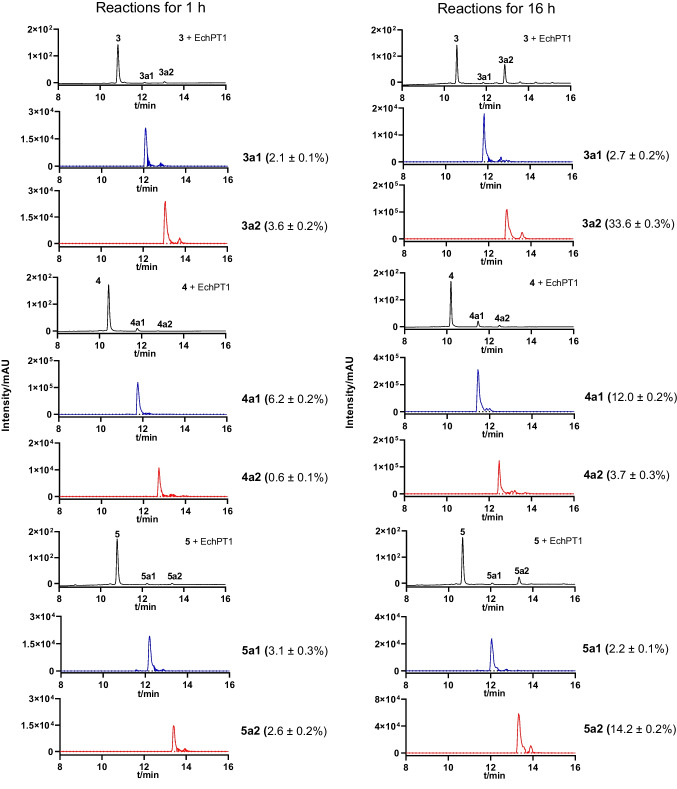
LCMS analysis of the acceptance of tetratryptomycins A–C (**3**–**5**) by EchPT1 for 1 and 16 h. UV absorptions at 280 nm are illustrated in black. The chromatograms depicted in blue and red refer to EICs of [M + H]^+^ of the monoprenylated at *m/z* 811.372 and those of the diprenylated products at *m/z* 879.434, respectively. A tolerance range of ± 0.005 was used for ion detection. All the assays were performed in duplicates. The conversion yields in percent are given in parenthesis after the product number as mean values. For better comparison, only the chromatograms between 8 and 16 min are illustrated

In comparison, cWW dimers like tetratryptomycins A–C (**3**–**5**) with the symmetrical C3-C3′ and the unsymmetrical C3-N1′ linkage were much better accepted, at least by two of the tested PTs. Incubation of **3**–**5** with the aforementioned five enzymes at 37 °C for 16 h and LCMS analysis showed that the C3-C3′ linked **3** and **5** were well consumed by EchPT1 with conversion yields for the sole products **3a2** and **5a2** at 33.6 ± 0.3 and 14.2 ± 0.2%, respectively. In the reaction mixture of the C3-N1′ linked **4** with EchPT1, the main product **4a1** with a conversion yield of 12.0 ± 0.2% was accompanied by the second product **4a2** with a conversion yield of 3.7 ± 0.3% (Fig. 3). Other four enzymes showed clearly lower catalytic activities toward **3**–**5** than EchPT1. FgaPT2_R244L showed a conversion yield of 8.8 ± 0.1% toward **4** and 6.5 ± 2.0% toward **5** for monoprenylated products. Almost no conversion of **3** and **4** was observed with FgaPT2_Y398F and 6-DMATS_Sa_ under the same conditions (Figs. S3–S5).

To detect monoprenylated products, we carried out incubations of **3**–**5** with EchPT1 in the presence of DMAPP for 1 h. As shown in Fig. 3, both monoprenylated (**3a1**, **4a1**, and **5a1**) with [M + H]^+^ ions at *m/z* 811.372 ± 0.005 and diprenylated products (**3a2**, **4a1**, and **5a2)** with [M + H]^+^ ions at *m/z* 879.434 ± 0.005 were clearly detected. Comparable product yields were calculated for the products of **3** and **5** in the range of 2.0–3.8%. The monoprenylated product **4a1** with a yield of 6.2 ± 0.2% was more accumulated in the reaction mixture of **4** than the diprenylated product **4a2** at 0.6 ± 0.1%. These results suggested that **3a1** and **5a1** were better accepted by EchPT1 than **4a1** for further prenylation.

**Fig. 4:**
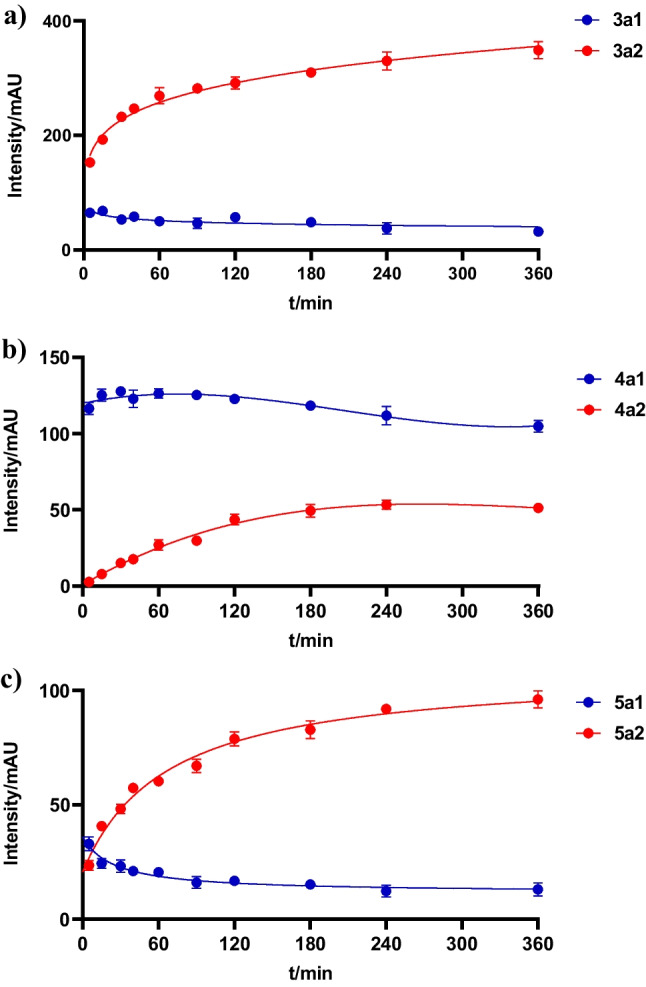
Time dependence of the mono- and diprenylated products formation in the EchPT1 reactions with **3**, **4**, and **5** and DMAPP. UV absorptions at 280 nm are illustrated. Error bars represent standard deviation from three independent experiments

To determine the relationship of mono- and diprenylated products in the reaction mixtures of the three cWW dimers with EchPT1, time dependence of their formation was determined. As shown in Fig. 4a, formation of **3a1** reached its maximum already in 5 min with a lower conversion yield than **3a2** and decreased slightly after that. In comparison, the formation of **3a2** increased continuously during the whole incubation process. Similar results were obtained for the formation of **5a1** and **5a2** with lower conversion yields than those for **3a1** and **3a2**. However, the maximum conversion yield of **5a1** is higher than that of **5a2** in 5 min (Fig. 4c). The product yield of **4a1** reached its maximum after 30 min, approximately ten-folds of that of **4a2** and then decreased slightly, while the formation of **4a2** started at a lower level and kept steady increasing. The amount of **4a1** is about two-folds of that of **4a2** after incubation for 6 h (Fig. 4b). These results correspond well to the results after incubation for 16 h (Fig. 3) and proved again **3a1** and **5a1** are much better substrates for EchPT1 than **4a1**.

### Prenylation of cWW dimers by EchPT1 and structural elucidation of the prenylated derivatives

To verify the structures of the four prenylated cWW dimers **3a2**, **4a1**, **4a2**, and **5a2**, the assays of **3**–**5** and DMAPP were scaled up to 25 ml and incubated for 16 h. After extraction with ethyl acetate and isolation on HPLC, these products with UV absorption maxima at approximately 224 and 284 nm were obtained in high purity (Fig. S9). High-resolution mass spectrometric data proved again the monoprenylation in **4a1** by detection of the [M + H]^+^ ion at *m/z* 811.3713, which is 68 dalton larger than that of the substrate **4**. In comparison, **3a2**, **4a2**, and **5a2** with [M + H]^+^ ions at *m/z* 879.434 ± 0.005 are diprenylated cWW dimers (Table S5).

The presence of the attached prenyl moieties in **3a2**, **4a1**, **4a2**, and **5a2** was also confirmed by comparing their NMR data (Table S1–S4) with those of **3**–**5** (Liu et al. 2020). In the ^1^H NMR spectra, the signals for indole H-19 (**3a2**, **4a1**, **4a2**, and **5a2**) and H-19′ (**3a2**, **4a2**, and **5a2**) disappeared. Instead, signals for one (**4a1**) or two (**3a2**, **4a2**, and **5a2**) reverse prenyl moieties can be observed by the characteristic chemical shifts and coupling patterns. A doublet of doublets with a chemical shift between 6.23 and 6.15 ppm was observed for H-28/H-28′. The coupling constants with the two protons at H-29/H-29′ also found as a doublet of doublets with a chemical shift around 5.11 to 5.00 ppm. The signals of the two methyl groups were detected at approximately 1.50 ppm (Figs. S7, S12, S17, and S22). These data indicate the attachment of the reverse prenyl moiety at C-19 (and C-19′) of the indole ring, which is also consistent with the EchPT1-catalyzed *C2*-prenylation at the indole ring for its natural substrate cWA.

**Fig. 5:**
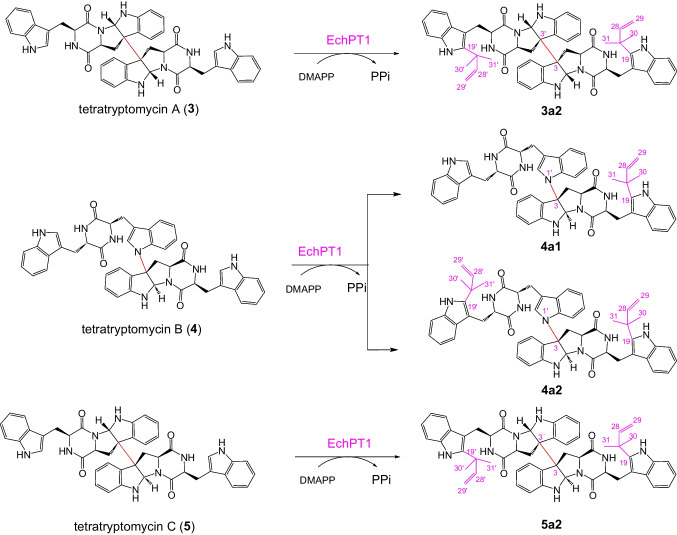
Prenylation of tetratryptomycins A–C by EchPT1 in the presence of DMAPP. See SI for carbon numbering

In the ^13^C NMR spectra, the signals of C-19/C-19′ at the indole ring were observed at δ_c_ 141.4–141.5ppm (Figs. S8, S13, S18, and S23), which were comparable with the NMR data of the *C2*-prenylated CDPs in the literature (Li et al. 2023). Clear long-range correlations between H-28 (H-30/H-31) and C-19 (**3a2**, **4a1**, **4a2**, and **5a2**) as well as H-28′ (H-30′/H-31′) and C-19′ (**3a2**, **4a2**, and **5a2**) were observed in the HMBC spectra, confirming the attachment of one or two reverse prenyl moieties at C-19 and C-19′ (Figs. S11, S16, S21, and S26). Taken together, their NMR data including ^1^H, ^13^C, ^1^H-^1^H COSY, HSQC, and HMBC proved unequivocally **4a1** as *C19*-prenylated tetratryptomycin B and **3a2**, **4a2**, and **5a2** as *C19,C19*′-diprenylated tetratryptomycins A, B, and C, respectively (Fig. 5). Prenylations at C-19/C-19′ do not change the stereochemistry of **3**–**5** and the absolute configurations of **3a2**, **4a2**, and **5a2** remain as those of their substrates (Liu et al. 2020).

### Antibacterial activities of the obtained prenylated cWW dimers

After structural elucidation, the obtained prenylated products were screened for their inhibitory activities against eight bacterial strains. No inhibition was detected for the isolated compounds **3a2**, **4a1**, **4a2**, and **5a2**.

### Determination of the kinetic parameters of EchPT1 toward tetratryptomycins and DMAPP

To further compare the catalytic efficiency of EchPT1 toward the dimeric derivatives **3**–**5** and DMAPP, kinetic parameters including Michaelis-Menten constants (*K*_*M*_) and turnover numbers (*k*_*cat*_) were determined for EchPT1. The most reactions followed the Michaelis-Menten kinetics, with the exception for **4a1** formation toward DMAPP and **4a2** formation toward **4** (Figs. S27–S30). Both of them fit well to a typical velocity equation with substrate inhibition (Figs. S28–S29). For the reactions of EchPT1 toward the prenyl acceptors **3**–**5**, the highest *K*_*M*_ at 0.25 mM was calculated for the formation of **4a1**, significantly higher than that of the natural substrate cWA at 0.09 mM. Interestingly, the Michaelis-Menten constants for the formation of **3a2**, **4a2**, and **5a2** at 0.06, 0.01, and 0.05 mM, respectively, are somewhat lower than that of cWA (Table 1). The *K*_*M*_ values of EchPT1 reaction toward DMAPP for the formation of the four products between 0.05 and 0.08 mM are also slightly lower than 0.18 mM in the presence of cWA. The turnover numbers from 0.002 to 0.14 s^−1^ and the *k*_*cat*_/*K*_*M*_ values from 286 to 1286 s^−1^ M^−1^ were determined in the range of EchPT1 reactions toward most CDPs (Wohlgemuth et al. 2018). The lowest *k*_*cat*_/*K*_*M*_ value of **4a2** at 25 s^−1^ M^−1^ is also in good consistence with the observed conversion yield depicted in Fig. 3.

**Table 1:** Kinetic parameters of EchPT1 toward **3**−**5** and DMAPP

Substrates	Products	Protein amount and incubation time	*K*_*M*_ (mM)	*k*_*cat*_ (s^−1^)	*k*_*cat*_/*K*_*M*_ (s^−1^ M^−1^)
**3**	**3a2**	7 μg, 30 min	0.06 ± 0.007	0.05 ± 0.0003	833
**4**	**4a1**	7 μg, 30 min	0.25 ± 0.03	0.14 ± 0.01	560
**4**	**4a2**	7 μg, 30 min	0.01 ± 0.001	0.006 ± 0.0006	600
**5**	**5a2**	7 μg, 20 min	0.05 ± 0.005	0.03 ± 0.001	600
DMAPP with **3**	**3a2**	7 μg, 30 min	0.05 ± 0.007	0.03 ± 0.002	600
DMAPP with **4**	**4a1**	7 μg, 30 min	0.07 ± 0.01	0.09 ± 0.01	1286
DMAPP with **4**	**4a2**	7 μg, 30 min	0.08 ± 0.008	0.002 ± 0.0002	25
DMAPP with **5**	**5a2**	7 μg, 20 min	0.07 ± 0.008	0.02 ± 0.001	286

Three independent experiments were carried out and standard deviations are given as ± values

## Discussion

Prenyltransferases of the DMATS superfamily are soluble proteins and can be easily overproduced in *Escherichia coli* (Fan et al. 2015; Winkelblech et al. 2015). They show high flexibility toward aromatic prenyl acceptors and therefore contribute significantly to structural diversity of small molecules. Numerous studies in the last years have demonstrated that such PTs can be utilized as biocatalysts for the target structures (Chen et al. 2017; Mori et al. 2016; Ostertag et al. 2020), since they can efficiently prenylate various natural and unnatural substrates including indole, naphthalene, xanthone, flavonoid, and cyclodipeptide derivatives (Fan et al. 2015; Winkelblech et al. 2015). Previous investigations revealed that prenylated products often exhibit improved interactions with proteins and biological membranes compared with the original precursors (Botta et al. 2009; Wollinsky et al. 2012).

As mentioned in the introduction, EchPT1 as a member of the echinulin biosynthetic pathway was first identified in *Aspergillus ruber* and catalyzes the reverse *C2*-prenylation of cWA at the indole ring, followed by additional prenylations with EchPT2 as the second PT of the pathway (Wohlgemuth et al. 2017). As most members of the DMATS superfamily, this enzyme also shows high flexibility toward aromatic substrates and accepted other cyclodipeptides for *C2*-prenylation (Wohlgemuth et al. 2018). Our recent study revealed that this enzyme is much more promiscuous than reported before. It can even accept already prenylated CDPs as substrates and catalyze reverse *C2*-prenylation at the indole nucleus (Li et al. 2023), which differs clearly from the nature’s strategy with the first prenylation at C-2 of unprenylated CDPs. These results encouraged us to expand the substrate spectrum of EchPT1.

As proof of concept, our main objective in this study was chemoenzymatic synthesis of prenylated dimeric CDPs in vitro. To the best of our knowledge, there is no report on dimeric CDP prenylating enzymes in the literature prior to this study. Four new cWW dimers with C19/C19′-prenylations on the indole ring were successfully obtained by prenylation of tetratryptomycins A–C with the promiscuous cWA prenyltransferase EchPT1 in the presence of DMAPP. Our results demonstrated that even complex molecules can be modified by known enzymes. Therefore, for designed small molecule modification, it is worth to test other available biocatalysts. As presented in this study, cWP-containing dimers were poor substrates of the tested PTs. In this case, it would be interesting to test other available PTs or to get mutants of the known enzymes as demonstrated by Xu for prenylation of biflavonoids (Xu et al. 2021).

### **Supplementary information**

ESM 1

## Data Availability

All data generated during this study are included in this published article and its supplementary information file.
