# Supplementary material for: Prenylation of dimeric cyclo-l-Trp-l-Trp by the promiscuous cyclo-l-Trp-l-Ala prenyltransferase EchPT1
Source: Appl Microbiol Biotechnol. 2023 Sep 15;107(22):6887–95. doi: 10.1007/s00253-023-12773-0 (PMC10589136; doi:10.1007/s00253-023-12773-0)
Supplement: Supplementary file 1 — ESM 1 [file 253_2023_12773_MOESM1_ESM.pdf]

Supporting Information

Prenylation of dimeric *cyclo*-L-Trp-L-Trp by the promiscuous *cyclo*-L-Trp-L-Ala prenyltransferase EchPT1

Wen Li,[a] Xiulan Xie,[b] Jing Liu,[a] Huili Yu[a] and Shu-Ming Li*[a]

[a] Institut für Pharmazeutische Biologie und Biotechnologie, Fachbereich Pharmazie, Philipps-Universität Marburg, Robert-Koch-Straße 4, 35037 Marburg, Germany.

[b] Fachbereich Chemie, Philipps-Universität Marburg, Hans-Meerwein-Straße 4, 35032 Marburg, Germany.

Corresponding to Shu-Ming Li, E-mail: shuming.li@staff.uni-marburg.de

Table of Contents

Supplementary Tables S4

Table S1. NMR data of compound **3a2** in DMSO-d_6 .... S4

Table S2. NMR data of compound **4a1** in DMSO-d_6 .... S6

Table S3. NMR data of compound **4a2** in DMSO-d_6 .... S8

Table S4. NMR data of compound **5a2** in DMSO-d_6 .... S10

Table S5. HRESIMS data of enzyme products. S12

Supplementary Figures S13

Figure S1. LCMS analysis of the acceptance of **1** by five prenyltransferases S13

Figure S2. LCMS analysis of the acceptance of **2** by five prenyltransferases S14

Figure S3. LCMS analysis of the acceptance of **3** by five prenyltransferases S15

Figure S4. LCMS analysis of the acceptance of **4** by five prenyltransferases S16

Figure S5. LCMS analysis of the acceptance of **5** by five prenyltransferases S17

Figure S6. UV spectra of the prenylated compounds **3a2**, **4a1**, **4a2**, and **5a2** S18

Figure S7. ^{1}H NMR spectrum of **3a2** in DMSO-d_6 (500 MHz) S19

Figure S8. ^{13}C NMR spectrum of **3a2** in DMSO-d_6 (125 MHz) S20

Figure S9. ^{1}H-^{1}H COSY spectrum of **3a2** in DMSO-d_6 .... S21

Figure S10. HSQC spectrum of **3a2** in DMSO-d_6 .... S22

Figure S11. HMBC spectrum of **3a2** in DMSO-d_6 .... S23

Figure S12. ^{1}H NMR spectrum of **4a1** in DMSO-d_6 (500 MHz) S24

Figure S13. ^{13}C NMR spectrum of **4a1** in DMSO-d_6 (125 MHz) S25

Figure S14. ^{1}H-^{1}H COSY spectrum of **4a1** in DMSO-d_6 .... S26

Figure S15. HSQC spectrum of **4a1** in DMSO-d_6 .... S27

Figure S16. HMBC spectrum of **4a1** in DMSO-d_6 .... S28

Figure S17. ^{1}H NMR spectrum of **4a2** in DMSO-d_6 (500 MHz) S29

Figure S18. ^{13}C NMR spectrum of **4a2** in DMSO-d_6 (125 MHz) S30

Figure S19. ^{1}H-^{1}H COSY spectrum of **4a2** in DMSO-d_6 .... S31

Figure S20. HSQC spectrum of **4a2** in DMSO-d_6 .... S32

Figure S21. HMBC spectrum of **4a2** in DMSO-d_6 .... S33

Figure S22. ^{1}H NMR spectrum of **5a2** in DMSO-d_6 (500 MHz) S34
Figure S23. ^{13}C NMR spectrum of **5a2** in DMSO-d_6 (125 MHz) S35
Figure S24. ^{1}H-^{1}H COSY spectrum of **5a2** in DMSO-d_6 .... S36
Figure S25. HSQC spectrum of **5a2** in DMSO-d_6 .... S37
Figure S26. HMBC spectrum of **5a2** in DMSO-d_6 .... S38
Figure S27. Determination of the kinetic parameters of EchPT1 for **3a2** formation toward **3** and DMAPP S39
Figure S28. Determination of the kinetic parameters of EchPT1 for **4a1** formation toward **4** and DMAPP S40
Figure S29. Determination of the kinetic parameters of EchPT1 for **4a2** formation toward **4** and DMAPP S41
Figure S30. Determination of the kinetic parameters of EchPT1 for **5a2** formation toward **5** and DMAPP S42

Supplementary Tables

Table S1. NMR data of compound **3a2** in DMSO-d_6

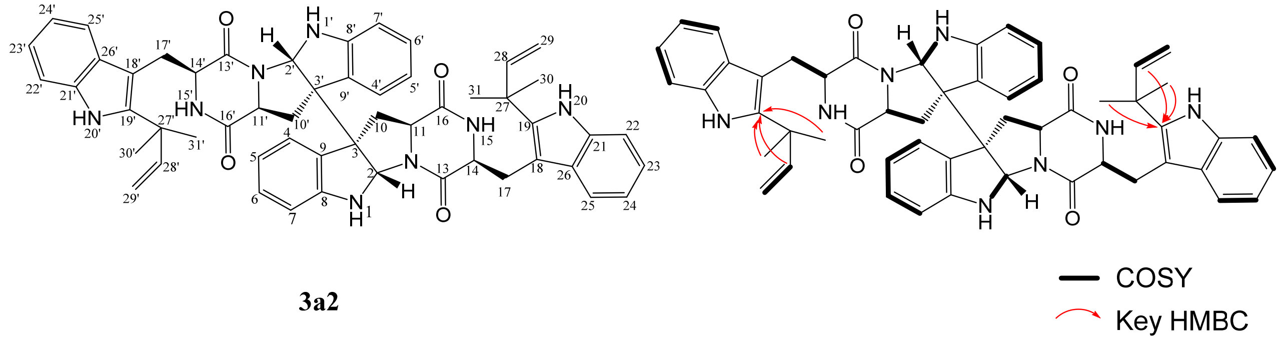

3a2

Pos.	δ_H (ppm) multi. *J* (Hz)	δ_C	COSY	HMBC
1	6.74 s	-	H-2	C-2, C-3, C-8, C-9
2	5.13 s	77.3		C-8
3	-	58.7		
4	7.26 d (7.6)	124.8	H-5	C-3, C-5, C-6
5	6.66 t (7.6)	117.4	H-4, H-6	C-7, C-9
6	7.03 t (7.6)	129.2	H-5	C-4, C-5, C-8
7	6.61 d (7.6)	109.0	H-6	C-5, C-9
8	-	151.2		
9	-	127.1		
10	2.53 m	38.9	H-11	C-2, C-3, C-9
	2.47 m			
11	3.88 dd (9.6, 6.8)	57.8	H-10	C-10, C-16
13	-	165.5		
14	4.29 dd (8.9, 4.7)	55.1	H-17	C-13, C-17
15	6.24 s	-		C-11, C-13, C-14, C-16
16	-	168.3		
17	3.42 dd (14.9, 4.7)	25.7	H-14	C-13, C-14, C-18, C-19, C-26
	2.89 (14.9, 8.9)			
18	-	104.3		
19	-	141.4		
20	10.65 s	-		C-18, C-19, C-26
21	-	134.8		
22	7.32 d (8.0)	111.3	H-23	C-24, C-26
23	7.06 t (7.4)	120.7	H-22	C-21, C-25
24	6.94 t (7.4)	118.6	H-25	C-22, C-26
25	7.43 d (8.0)	117.8	H-24	C-18, C-21, C-23, C-26
26	-	128.7		
27	-	40.4		
28	6.17 dd (17.4, 10.5)	146.4	H-29	C-19, C-27, C-30, C-31

(Continued on next page)

Table S1 (Continued)

Pos.	δH (ppm) multi. *J* (Hz)	δC	COSY	HMBC
29	5.05 dd (17.4, 1.3)	111.3	H-28	C-27, C-28, C-30, C-31
	5.02 dd (10.5, 1.3)			
30	1.48 s	27.9		C-19, C-27, C-28, C-29, C-31
31	1.47 s	27.8		C-19, C-27, C-28, C-29, C-30
1′	6.74 s	-	H-2′	C-2′, C-3′, C-8′, C-9′
2′	5.13 s	77.3		C-8′
3′	-	58.7		
4′	7.26 d (7.6)	124.8	H-5′	C-3′, C-5′, C-6′
5′	6.66 t (7.6)	117.4	H-4′, H-6′	C-7′, C-9′
6′	7.03 t (7.6)	129.2	H-5′	C-4′, C-5′, C-8′
7′	6.61 d (7.6)	109.0	H-6′	C-5′, C-9′
8′	-	151.2		
9′	-	127.1		
10′	2.53 m	38.9	H-11′	C-2′, C-3′, C-9′
	2.47 m			
11′	3.88 dd (9.6, 6.8)	57.8	H-10′	C-10′, C-16′
13′	-	165.5		
14′	4.29 dd (8.9, 4.7)	55.1	H-17′	C-13′, C-17′
15′	6.24 s	-		C-11′, C-13′, C-14′, C-16′
16′	-	168.3		
17′	3.42 dd (14.9, 4.7)	25.7	H-14′	C-13′, C-14′, C-18′, C-19′, C-26′
	2.89 (14.9, 8.9)			
18′	-	104.3		
19′	-	141.4		
20′	10.65 s	-		C-18′, C-19′, C-26′
21′	-	134.8		
22′	7.32 d (8.0)	111.3	H-23′	C-24′, C-26′
23′	7.06 t (7.4)	120.7	H-22′	C-21′, C-25′
24′	6.94 t (7.4)	118.6	H-25′	C-22′, C-26′
25′	7.43 d (8.0)	117.8	H-24′	C-18′, C-21′, C-23′, C-26′
26′	-	128.7		
27′	-	40.4		
28′	6.17 dd (17.4, 10.5)	146.4	H-29′	C-19′, C-27′, C-30′, C-31′
29′	5.05 dd (17.4, 1.3)	111.3	H-28′	C-27′, C-28′, C-30′, C-31′
	5.02 dd (10.5, 1.3)			
30′	1.48 s	27.9		C-19′, C-27′, C-28′, C-29′, C-31′
31′	1.47 s	27.8		C-19′, C-27′, C-28′, C-29′, C-30′

Table S2. NMR data of compound **4a1** in DMSO-d_6

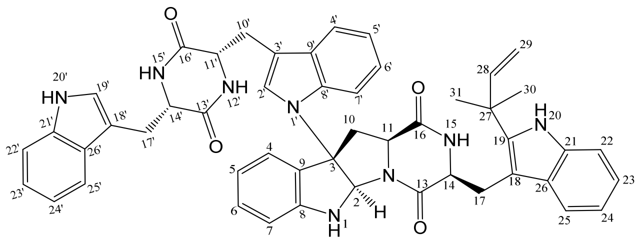

4a1

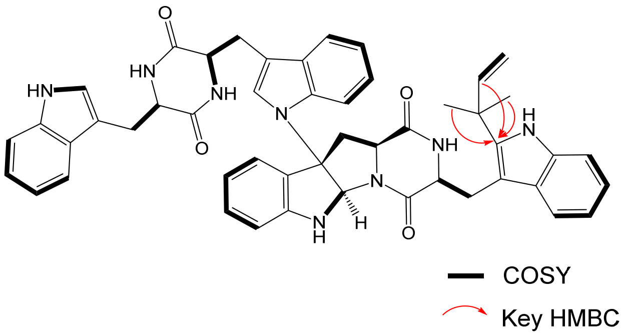

Pos.	δH (ppm) multi. *J* (Hz)	δC	COSY	HMBC
1	7.27 d (3.9)	-	H-2	C-2, C-3, C-4, C-9
2	5.78 d (3.9)	81.5	H-1	C-3, C-8, C-10, C-11
3	-	73.5		
4	6.71 d (8.3)	110.1	H-5, H-6	C-5, C-8, C-9
5	6.53 t (7.5)	118.0	H-4, H-6	C-4, C-7, C-9
6	7.12 t (7.5)	129.7	H-4, H-5	C-4, C-7, C-8
7	6.71 d (8.3)	122.2	H-6	C-5, C-8, C-9
8	-	148.0		
9	-	128.6		
10	3.45 dd (14.3, 5.8)<br>2.22 dd (14.3, 12.2)	38.9	H-11	C-2, C-3, C-9, C-11, C-16
11	4.65 dd (11.5, 5.8)	57.5	H-10	C-10, C-16
12	-	-		
13	-	167.9		
14	4.41 dd (8.3, 5.5)	55.5	H-17	C-13, C-17, C-18
15	6.67 s	-		C-11, C-13, C-14, C-17
16	-	169.2		
17	3.50 dd (15.3, 5.5)<br>3.02 m	24.7	H-14	C-13, C-14, C-18, C-19, C-26
18	-	104.4		
19	-	141.5		
20	10.68 s	-		C-18, C-19, C-21, C-26, C-a
21	-	136.0		
22	7.33 d (8.1)	111.6	H-23	C-24, C-26
23	7.11 t (7.8)	120.7	H-22	C-21, C-25
24	6.97 t (7.8)	118.7	H-25	C-22, C-26
25	7.54 d (8.1)	118.2	H-24	C-18, C-21, C-23, C-26
26	-	128.7		
27	-	40.4		
28	6.23 dd (17.4, 10.5)	146.3	H-c	C-19, C-a, C-d, C-e

(Continued on next page)

Table S2 (Continued)

Pos.	δ_H (ppm) multi. *J* (Hz)	δ_C	COSY	HMBC
29	5.11 d (17.4)	111.5	H-b	C-a, C-b
	5.08 d (10.5)			
30	1.52 s	27.8		C-a, C-b, C-c, C-e, C-19
31	1.50 s	27.9		C-a, C-b, C-c, C-d, C-19
1′	-	-		
2′	6.41 s	124.7		C-3, C-3′, C-7′, C-8′, C-9′, C-10′
3′	-	109.1		
4′	7.19 d (8.2)	118.7	H-5′	C-3′, C-6′, C-8′, C-9′
5′	6.93 t (7.6)	119.1	H-4′	C-6′, C-7′, C-9′
6′	6.87 t (7.6)	120.9	H-7′	C-5′, C-8′
7′	6.49 d (8.2)	111.8	H-6′	C-5′, C-9′
8′	-	134.8		
9′	-	129.2		
10′	2.61 dd (14.1, 2.8)	30.3	H-11′	C-2′, C-3′, C-9′, C-11′, C-16′
	1.16 dd (14.1, 9.9)			
11′	3.67 td (9.7, 2.8)	54.5	H-10′	C-3′, C-13′
12′	7.59 d (2.1)		H-11′	C-14′, C-16′
13′	-	166.6		
14′	4.05 d (2.3)	55.5	H-17′	C-16′, C-18′
15′	8.13 d (1.6)		H-14′	C-11′, C-13′
16′	-	166.9		
17′	3.02 m	29.8	H-14′	C-13′, C-14′, C-16′, C-18′, C-19′, C-22′
	2.87 dd (14.2, 4.3)	-		
18′	-	108.6		
19′	6.89 d (1.9)	124.9	H-20′	C-18′, C-21′, C-26′
20′	10.98 s	-	H-19′	C-18′, C-19′, C-21′, C-26′
21′		135.1		
22′	7.36 d (8.1)	111.0	H-23′	C-23′, C-26′
23′	7.04 t (7.1)	118.5	H-22′, H-24′	C-21′, C-22′
24′	7.03 t (7.1)	121.0	H-25′	C-21′, C-22′, C-23′, C-26′
25′	7.52 d (8.1)	119.2	H-24′	C-18′, C-21′, C-22′, C-24′, C-26′
26′	-	127.7		

Table S3. NMR data of compound **4a2** in DMSO-d_6

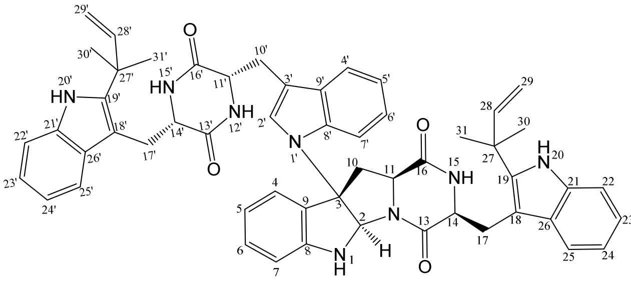

4a2

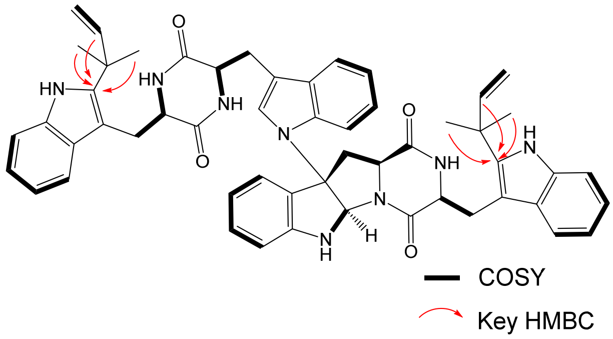

Pos.	δH (ppm) multi. *J* (Hz)	δC	COSY	HMBC
1	7.30 d (4.0)	-	H-2	C-2, C-3, C-5, C-9
2	5.82 d (4.0)	81.6	H-1	C-3, C-8, C-10, C-11
3	-	73.6		
4	6.69 d (8.2)	122.3	H-5	C-3, C-6, C-8
5	6.45 td (7.6, 1.3)	118.2	H-4, H-6	C-4, C-7, C-9
6	7.11 td (7.6, 1.3)	129.7	H-5, H-7	C-4, C-5, C-7, C-8
7	6.72 d (8.2)	110.1	H-6	C-5, C-9
8	-	148.0		
9	-	128.6		
10	3.55 dd (14.5, 6.2)<br>2.26 dd (14.5, 11.8)	39.0	H-11	C-2, C-3, C-9, C-11, C-16
11	4.76 dd (11.8, 6.2)	57.5	H-10	C-10, C-16
12	-			
13	-	167.9		
14	4.44 dd (8.6, 5.3)	55.4	H-17	C-13, C-17, C-18
15	6.64 s	-		C-11, C-13, C-14, C-17
16	-	169.1		
17	3.50 dd (14.8, 5.3)<br>3.02 dd (14.8, 9.3)	24.7	H-14	C-13, C-14, C-18, C-19, C-26
18	-	104.9		
19	-	141.5		
20	10.51 s	-		C-18, C-19, C-21, C-24, C-26
21	-	134.9		
22	7.28 d (8.4)	110.6	H-23	C-24, C-26
23	7.03 td (7.5, 1.3)	120.4	H-22, H-24	
24	6.81 td (7.5, 1.3)	118.3	H-23, H-25	C-22, C-23, C-25, C-26
25	7.05 d (8.4)	118.6	H-24	C-18, C-21, C-24, C-26
26	-	129.2		
27	-	40.4		
28	6.22 dd (17.4, 10.5)	146.5	H-29	C-19, C-27, C-30, C-31

(Continued on next page)

Table S3 (Continued)

Pos.	δ_H (ppm) multi. *J* (Hz)	δ_C	COSY	HMBC
29	5.09 dd (17.4, 1.3)	111.5	H-28	C-19, C-27, C-28
	5.07 dd (10.5, 1.3)			
30	1.51 s	28.0		C-19, C-27, C-28, C-29, C-31
31	1.49 s	27.9		C-19, C-27, C-28, C-29, C-30
1′	-	-		
2′	7.09 d (1.3)	125.0		C-3′, C-8′, C-9′
3′	-	109.2		
4′	7.48 d (8.2)	118.9	H-5′	C-3′, C-6′, C-8′, C-9′
5′	7.04 td (7.5, 1.3)	120.7	H-4′	C-7′, C-9′
6′	6.90 td (7.5, 1.3)	121.3	H-5′, H-7′	C-4′, C-8′
7′	6.54 d (8.2)	111.9	H-6′	C-5′, C-9′
8′	-	134.7		
9′	-	129.4		
10′	2.45 dd (14.6, 8.8)	38.9	H-11′	C-2′, C-3′, C-9′
	3.02 dd (14.6, 9.3)			
11′	3.94 dd (8.8, 3.5)	55.1	H-10′	C-10′, C-13′
12′	7.62 d (3.0)	-	C-11′	C-14′, C-16′
13′	-	166.7		
14′	3.94 dd (7.8, 3.9)	56.3	H-17′	C-16′, C-18′
15′	8.05 d (2.7)	-	H-14′	C-11′, C-13′
16′	-	167.4		
17′	3.28 dd (14.5, 3.9)	30.3	H-14′	C-13′, C-14′, C-18′, C-19′, C-26′
	2.77 dd (14.5, 7.8)			
18′	-	104.4		
19′	-	141.4		
20′	10.67 s	-		C-18′, C-19′, C-21′, C-24′, C-26′
21′	-	134.8		
22′	7.34 d (8.0)	111.1	H-23′	C-23′, C-26′
23′	7.00 td (7.5, 1.3)	119.4	H-22′, H-24′	C-21′, C-25′
24′	6.96 td (7.5, 1.3)	118.7	H-23′, H-25′	C-22′, C-25′, C-26
25′	7.52 d (8.0)	118.0	H-24′	C-18′, C-21′, C-23′, C-26
26′	-	128.6		
27′	-	40.4		
28′	6.15 dd (17.4, 10.5)	146.3	H-29′	C-19′, C-27′, C-30′, C-31′
29′	5.05 dd (17.4, 1.3)	111.2	H-28′	C-19′, C-27′, C-28′
	5.00 dd (10.5, 1.3)			
30′	1.46 s	27.9		C-19′, C-27′, C-28′, C-29′, C-31′
31′	1.46 s	27.8		C-19′, C-27′, C-28′, C-29′, C-30′

Table S4. NMR data of compound **5a2** in DMSO-d_6

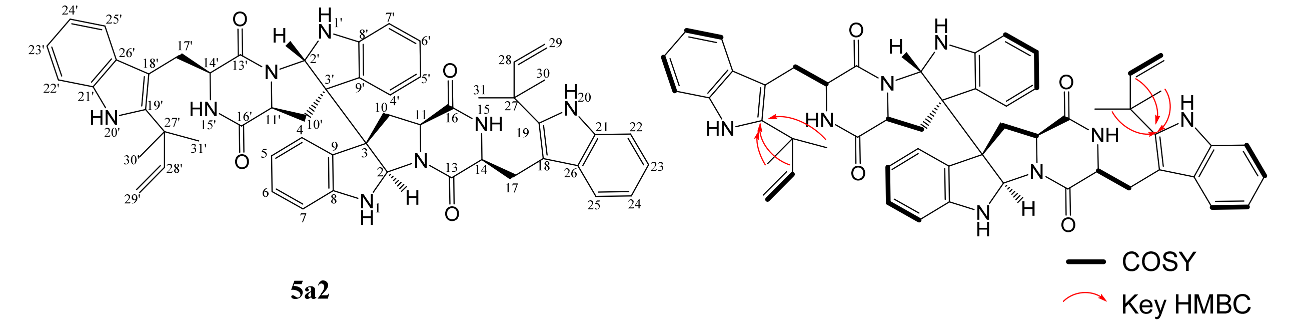

Pos.	δH (ppm) multi. *J* (Hz)	δC	COSY	HMBC
1	6.74 s	-		
2	5.55 s	75.9 [a]		
3	-	63.1		
4	6.52 m	123.9		110.1
5	6.52 m	117.9	H-6	118.0
6	6.99 m	129.1	H-5, H-7	129.7
7	6.52 m	108.6 [a]	H-6	122.2
8	-	151.3		
9	-	130.5		
10	2.55 m	39.0	H-11	
	2.30 m			
11	3.94 dd (9.9, 6.4)	57.1	H-10	
13	-	168.5		
14	4.33 dd (9.0, 4.3)	55.2	H-17	
15	6.23 s	-		C-11, C-13
16	-	169.4		
17	3.53 dd (15.4, 4.3)	24.7	H-14	C-13, C-14, C-18, C-19, C-26
	2.90 dd (15.4, 9.6)			
18	-	104.6		
19	-	141.5		
20	10.68 s	-		C-18, C-19, C-21, C-26
21	-	134.9		
22	7.34 d (7.9)	111.1	H-23	C-24, C-26
23	7.04 t (7.5)	120.8	H-22	C-21, C-25
24	6.94 t (7.5)	118.7	H-25	C-22, C-26
25	7.49 d (7.9)	117.9	H-24	C-18, C-21, C-23, C-26
26	-	128.8		
27	-	40.4		
28	6.17 dd (17.3, 10.5)	146.4	H-29	C-19, C-27, C-30, C-31
29	5.03 d (17.3)	111.4	H-28	C-27, C-28
	5.01 d (10.5)			

(Continued on next page)

Table S4 (Continued)

Pos.	δH (ppm) multi. *J* (Hz)	δC	COSY	HMBC
30	1.49 s	27.9		C-19, C-27, C-28, C-29, C-31
31	1.49 s	27.9		C-19, C-27, C-28, C-29, C-30
1′	-	-		
2′	5.41 s	72.5		
3′	-	59.9		
4′	6.52 m	123.8		
5′	6.52 m	117.8	H-6′	
6′	6.99 m	128.5	H-5′, H-7′	
7′	6.52 m	108.3 [a]	H-6′	
8′	-	149.1		
9′	-	127.7		
10′	3.43 m	38.9	H-11′	
	3.28 dd (11.1, 5.7)			
11′	3.46 m	57.7	H-10′	
13′	-	165.2		
14′	4.33 dd (9.0, 4.3)	55.9	H-17′	
15′	6.17 s	-		
16′	-	167.9		
17′	3.53 dd (15.4, 4.3)	26.3	H-14′	C-13′, C-14′, C-18′, C-19′, C-26′
	2.99 dd (15.4, 9.6)			
18′	-	104.2		
19′	-	141.4		
20′	10.66 s	-		C-18′, C-19′, C-21 ′, C-26 ′
21′	-	134.8		
22′	7.34 d (7.9)	111.1	H-23′	C-24′, C-26′
23′	7.04 t (7.5)	120.8	H-22′	C-21′, C-25′
24′	6.94 t (7.5)	118.7	H-25′	C-22′, C-26′
25′	7.44 d (7.9)	117.8	H-24′	C-18′, C-21′, C-23′, C-26′
26′	-	128.6		
27′	-	40.4		
28′	6.17 dd (17.3, 10.5)	146.3	H-29′	C-19′, C-27′, C-30′, C-31′
29′	5.03 d (17.3)	111.3	H-28′	C-27′, C-28′
	5.01 d (10.5)			
30′	1.48 s	27.8		C-19′, C-27′, C-28′, C-29′, C-31′
31′	1.48 s	27.8		C-19′, C-27′, C-28′, C-29′, C-30′

[a] Signals were only detected in HSQC

Table S5. HRESIMS data of enzyme products

product	chemical formula	HRESIMS data		
		**measured $[M + H]^+$**	**calculated $[M + H]^+$**	**deviation [ppm]**
**3a2**	$C_{54}H_{54}N_8O_4$	879.4335	879.4341	1.38
**4a1**	$C_{49}H_{46}N_8O_4$	811.3713	811.3715	0.25
**4a2**	$C_{54}H_{54}N_8O_4$	879.4343	879.4341	0.23
**5a2**	$C_{54}H_{54}N_8O_4$	879.4337	879.4341	0.45

Supplementary Figures

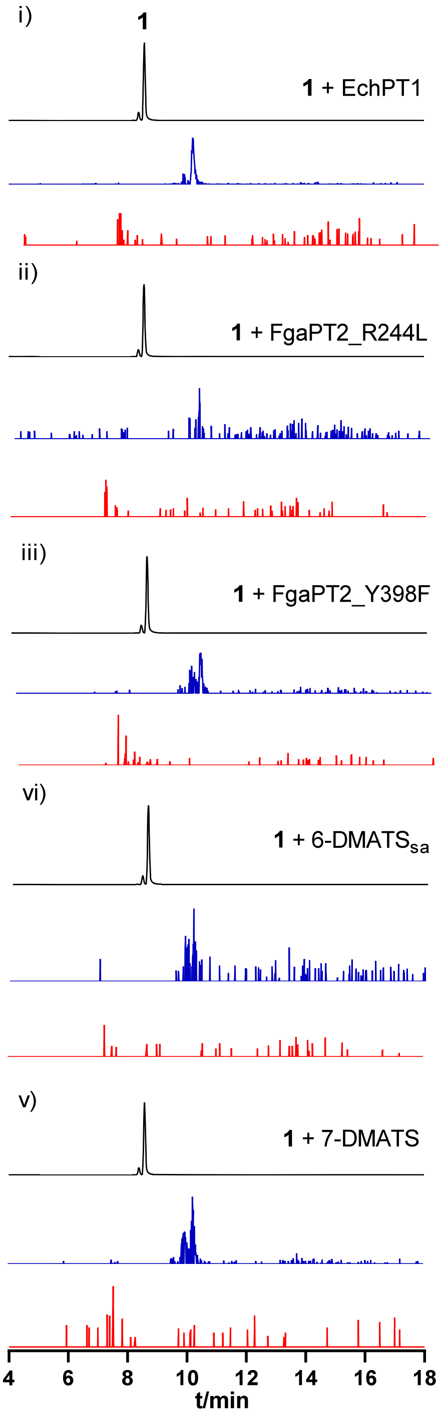

Figure S1. LCMS analysis of the acceptance of aspergilazine A (**1**) by EchPT1, FgaPT2_R244L, FgaPT2_Y398F, 6-DMATSSa, and 7-DMATS. UV absorptions at 280 nm are illustrated in black. The chromatograms depicted in blue and red refer to EICs of $[M + H]^+$ for the monoprenylated at *m/z* 635.789 and those of diprenylated products at *m/z* 702.900, respectively. A tolerance range of ± 0.005 was used for ion detection.

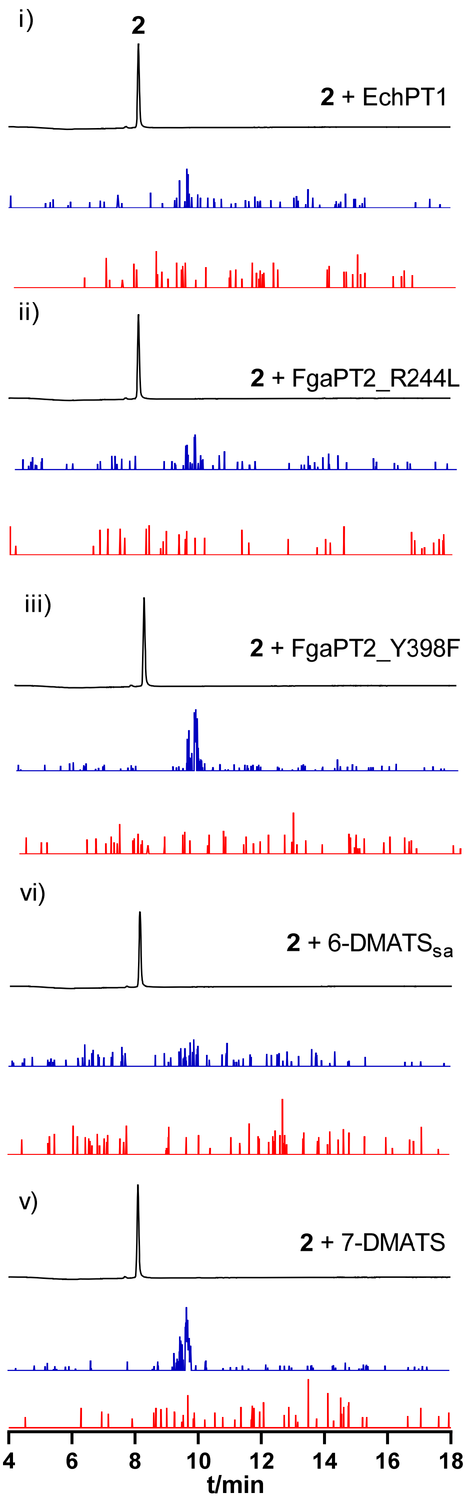

Figure S2. LCMS analysis of the acceptance of naseseazine A (**2**) by EchPT1, FgaPT2_R244L, FgaPT2_Y398F, 6-DMATSsa, and 7-DMATS. UV absorptions at 280 nm are illustrated in black. The chromatograms depicted in blue and red refer to EICs of $[M+H]^+$ for the monoprenylated at *m/z* 607.735 and those of diprenylated products at *m/z* 674.846. A tolerance range of ± 0.005 was used for ion detection.

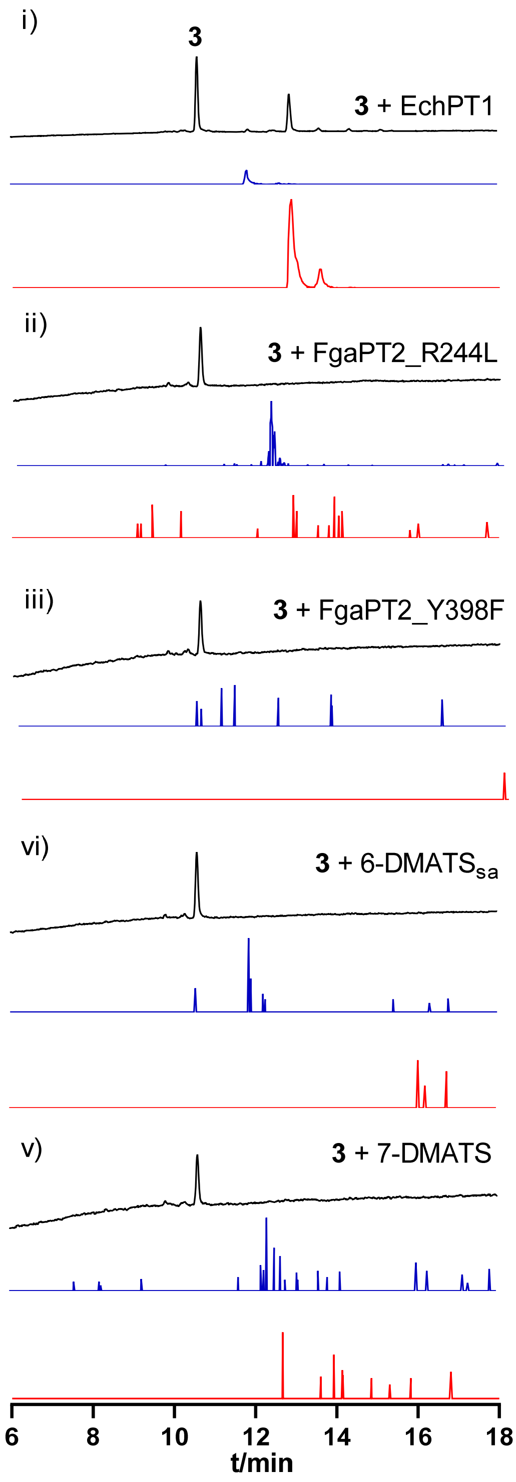

Figure S3. LCMS analysis of the acceptance of tetratryptomycin A (**3**) by EchPT1, FgaPT2_R244L, FgaPT2_Y398F, 6-DMATS$_{Sa}$, and 7-DMATS. UV absorptions at 280 nm are illustrated in black. The chromatograms depicted in blue and red refer to EICs of $[M + H]^+$ for the monoprenylated at *m/z* 811.372 and those of diprenylated products at *m/z* 879.434. A tolerance range of ± 0.005 was used for ion detection.

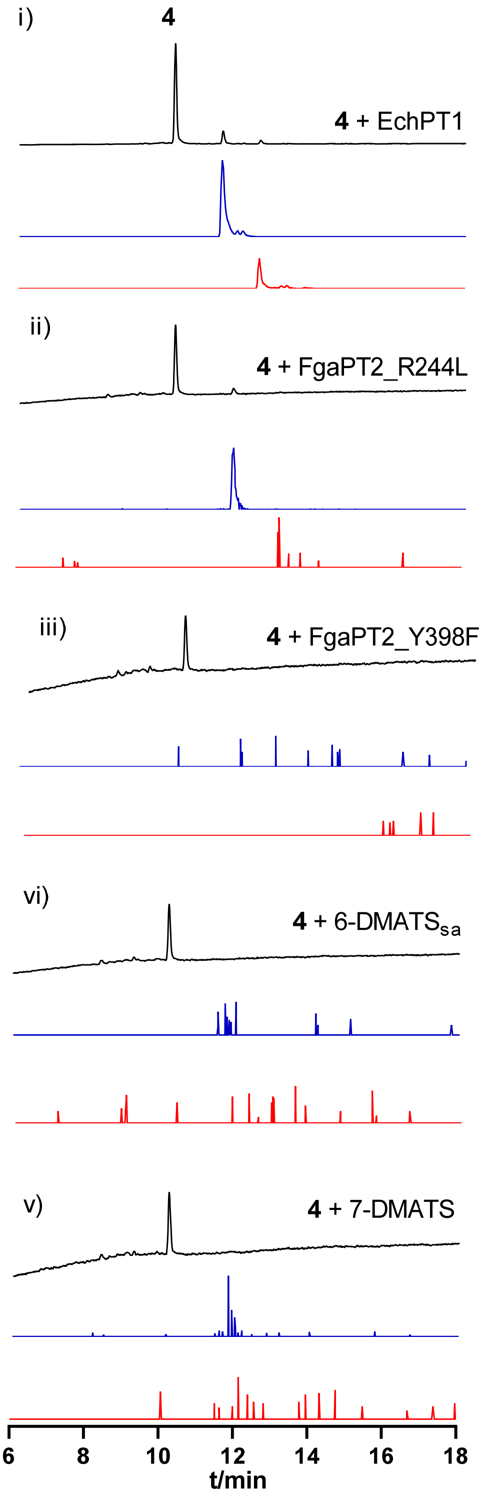

Figure S4. LCMS analysis of the acceptance of tetratryptomycin B (**4**) by EchPT1, FgaPT2_R244L, FgaPT2_Y398F, 6-DMATSSa, and 7-DMATS. UV absorptions at 280 nm are illustrated in black. The chromatograms depicted in blue and red refer to EICs of $[M + H]^+$ for the monoprenylated at *m/z* 811.372 and those of diprenylated products at *m/z* 879.434. A tolerance range of ± 0.005 was used for ion detection.

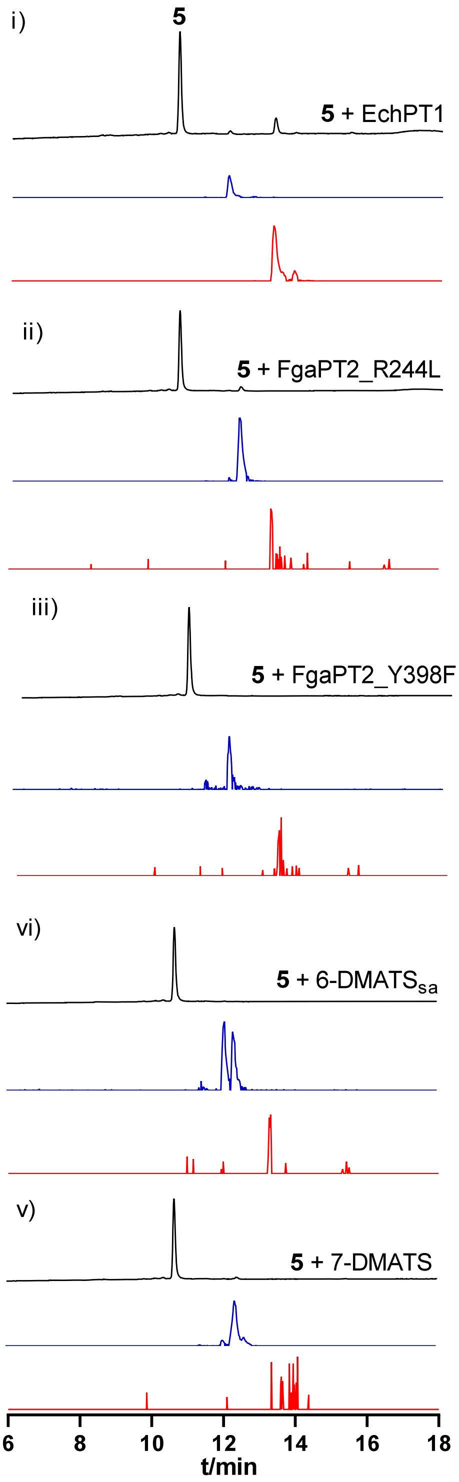

Figure S5. LCMS analysis of the acceptance of tetratryptomycin C (**5**) by EchPT1, FgaPT2_R244L, FgaPT2_Y398F, 6-DMATSsa, and 7-DMATS. UV absorptions at 280 nm are illustrated in black. The chromatograms depicted in blue and red refer to EICs of $[M + H]^+$ for the monoprenylated at *m/z* 811.372 and those of diprenylated products at *m/z* 879.434. A tolerance range of ± 0.005 was used for ion detection.

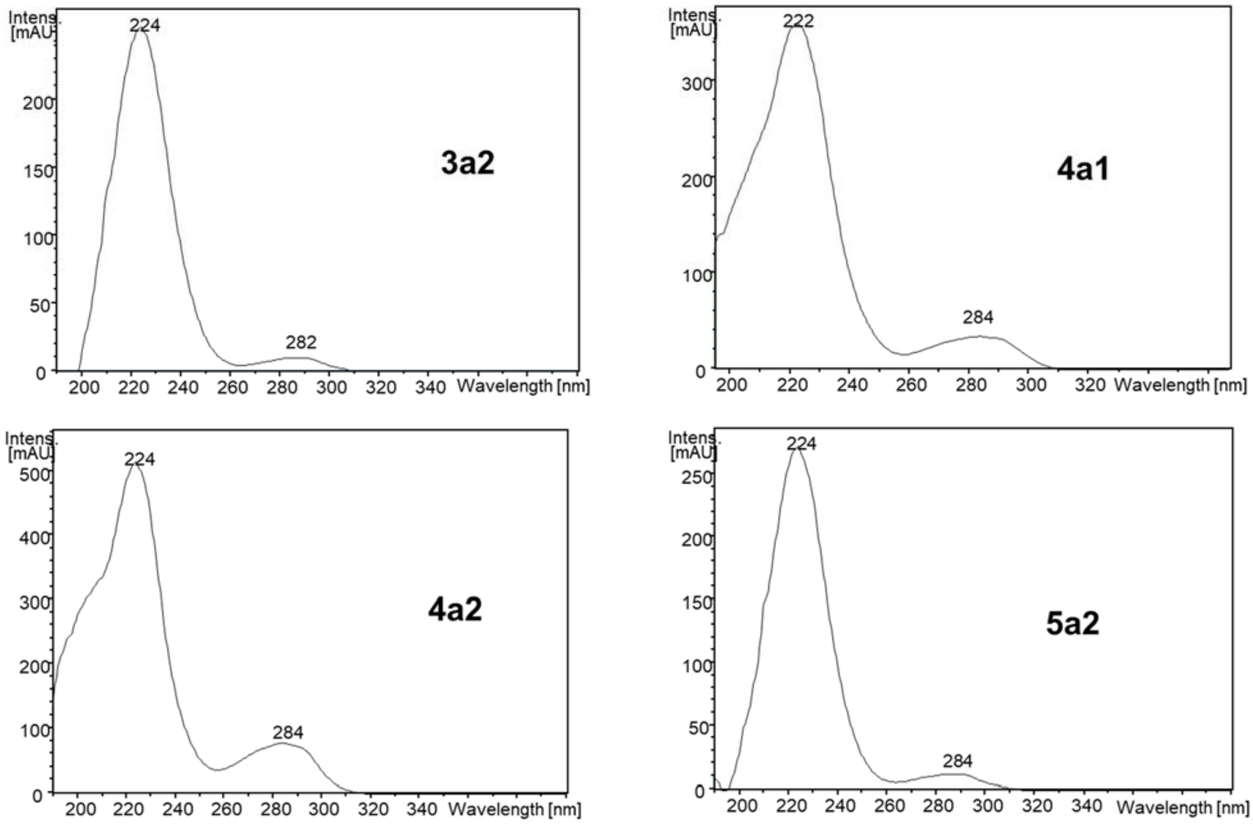

Figure S6. UV spectra of the prenylated compounds **3a2**, **4a1**, **4a2**, and **5a2**.

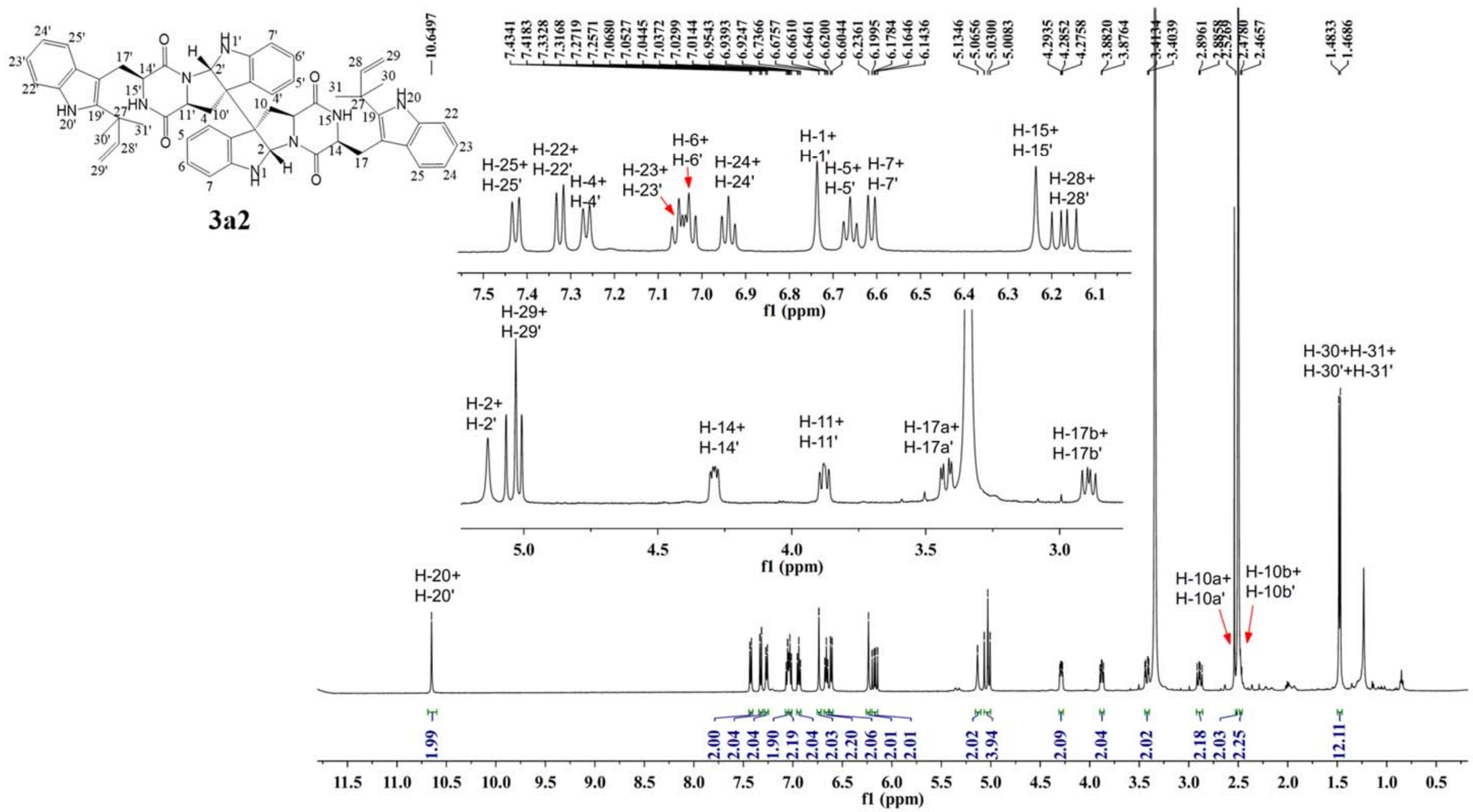

Figure S7. ^{1}H NMR spectrum of **3a2** in DMSO-d_6 (500 MHz).

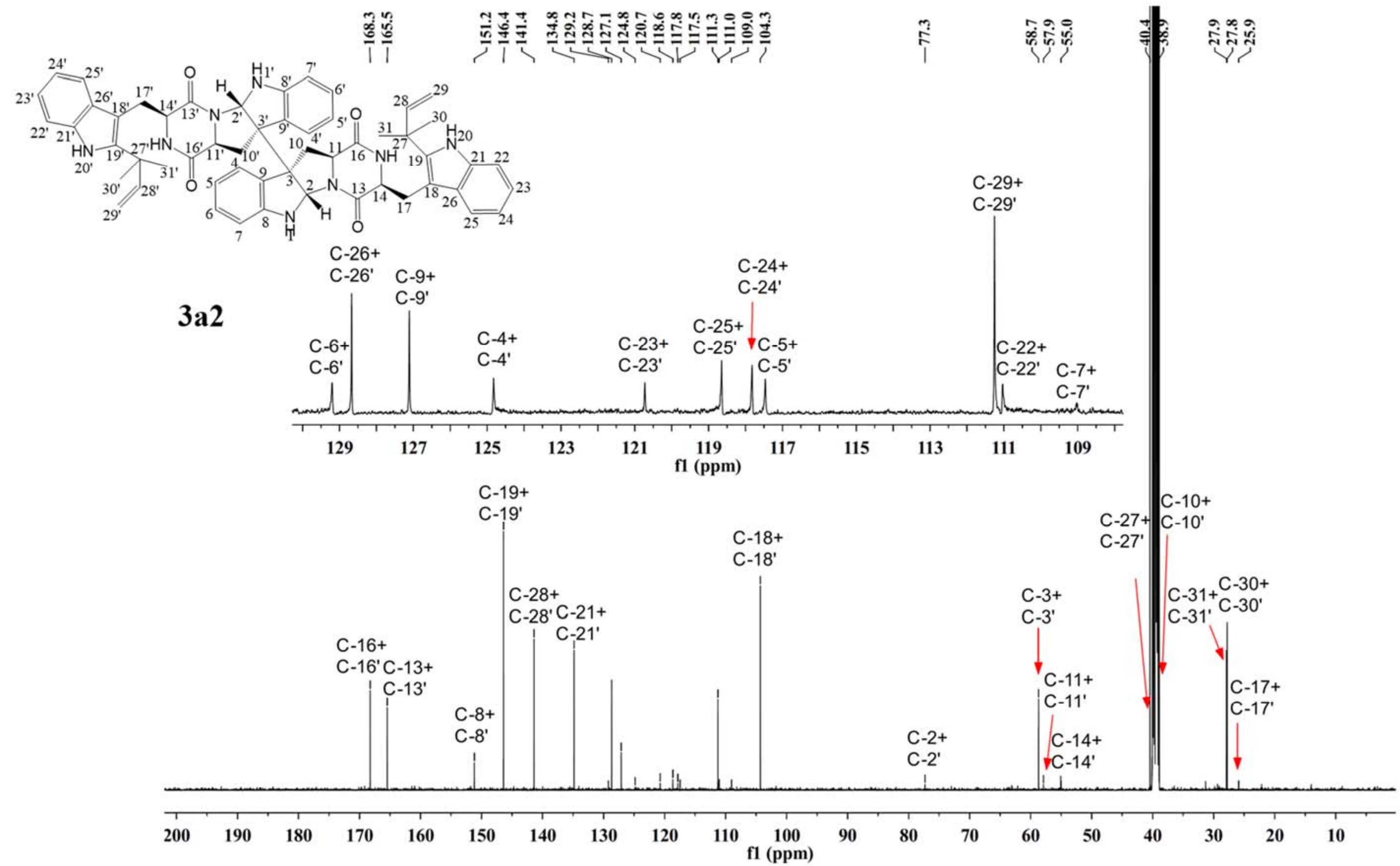

Figure S8. ^{13}C NMR spectrum of **3a2** in DMSO-d_6 (125 MHz).

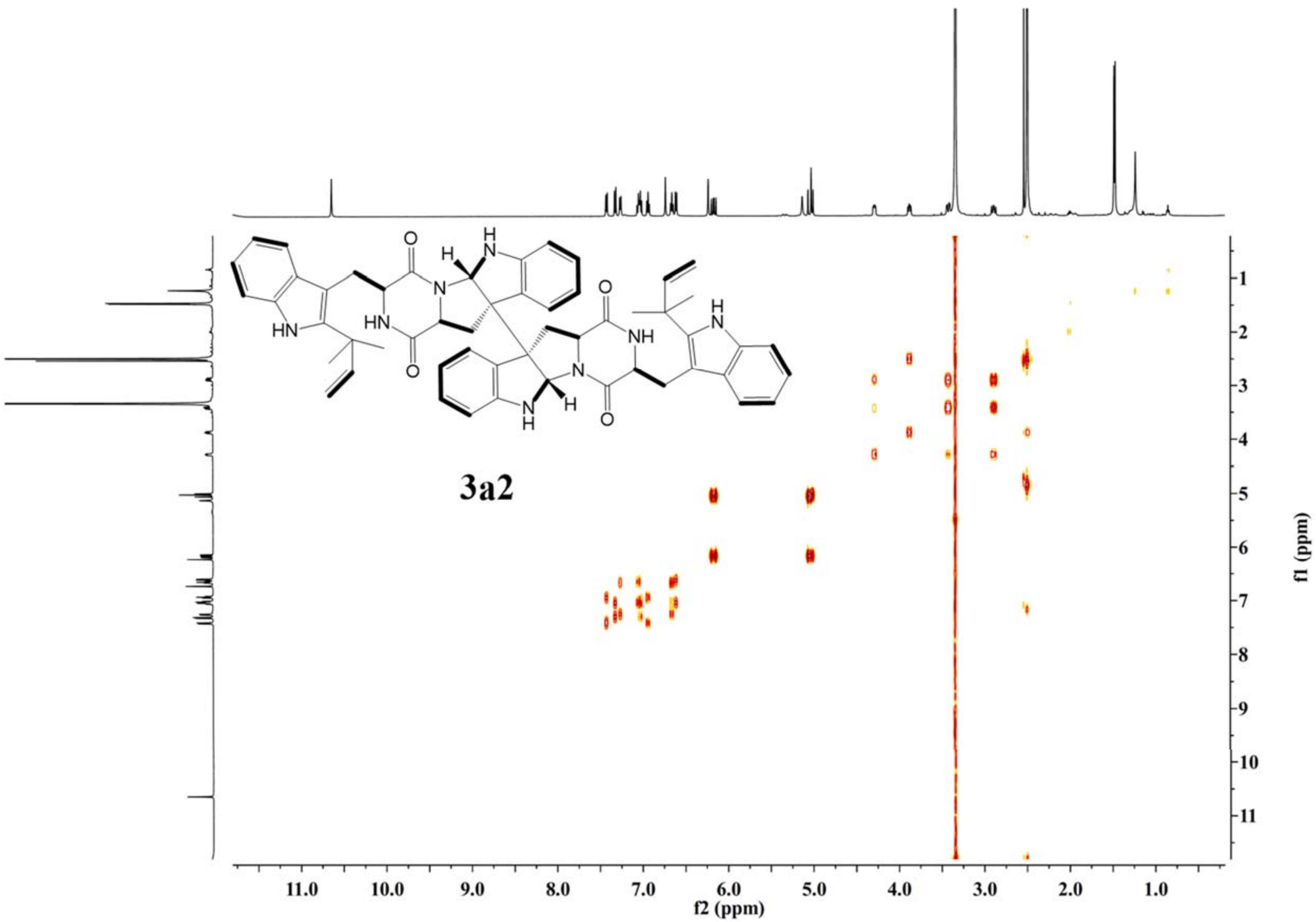

Figure S9. ^1H-^1H COSY spectrum of **3a2** in DMSO-d_6.

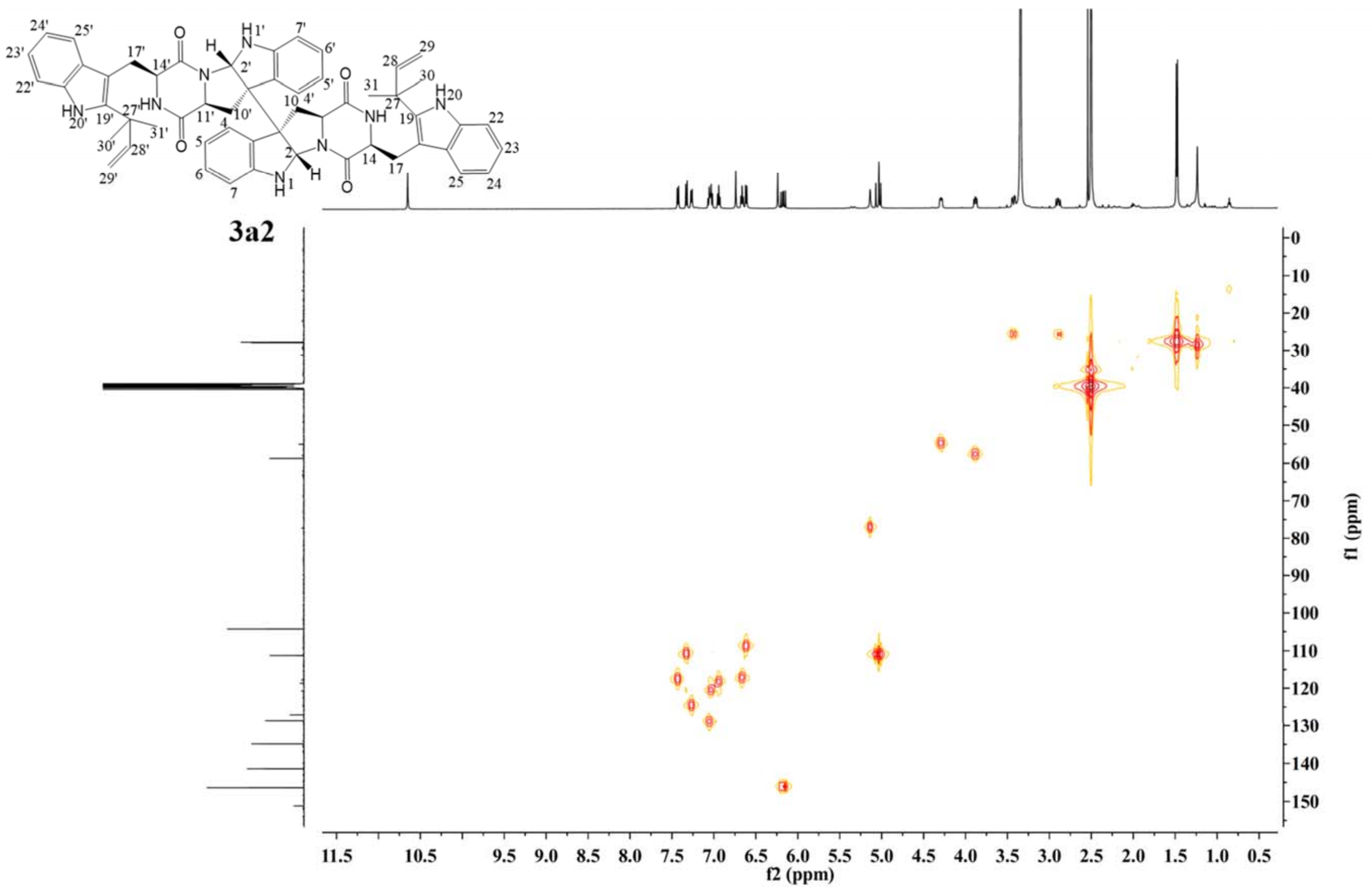

Figure S10. HSQC spectrum of **3a2** in DMSO-d_6.

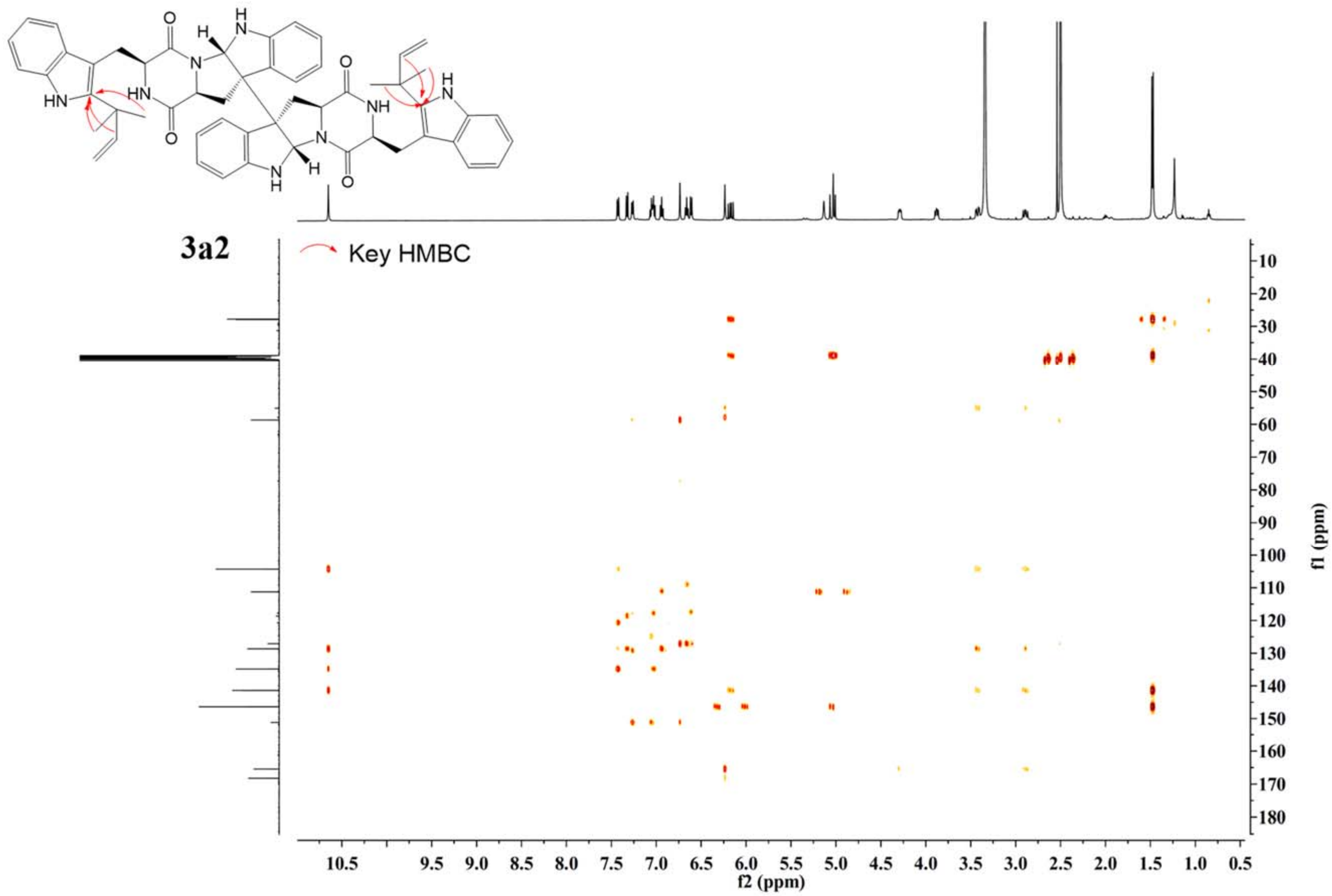

Figure S11. HMBC spectrum of **3a2** in DMSO-d_6.

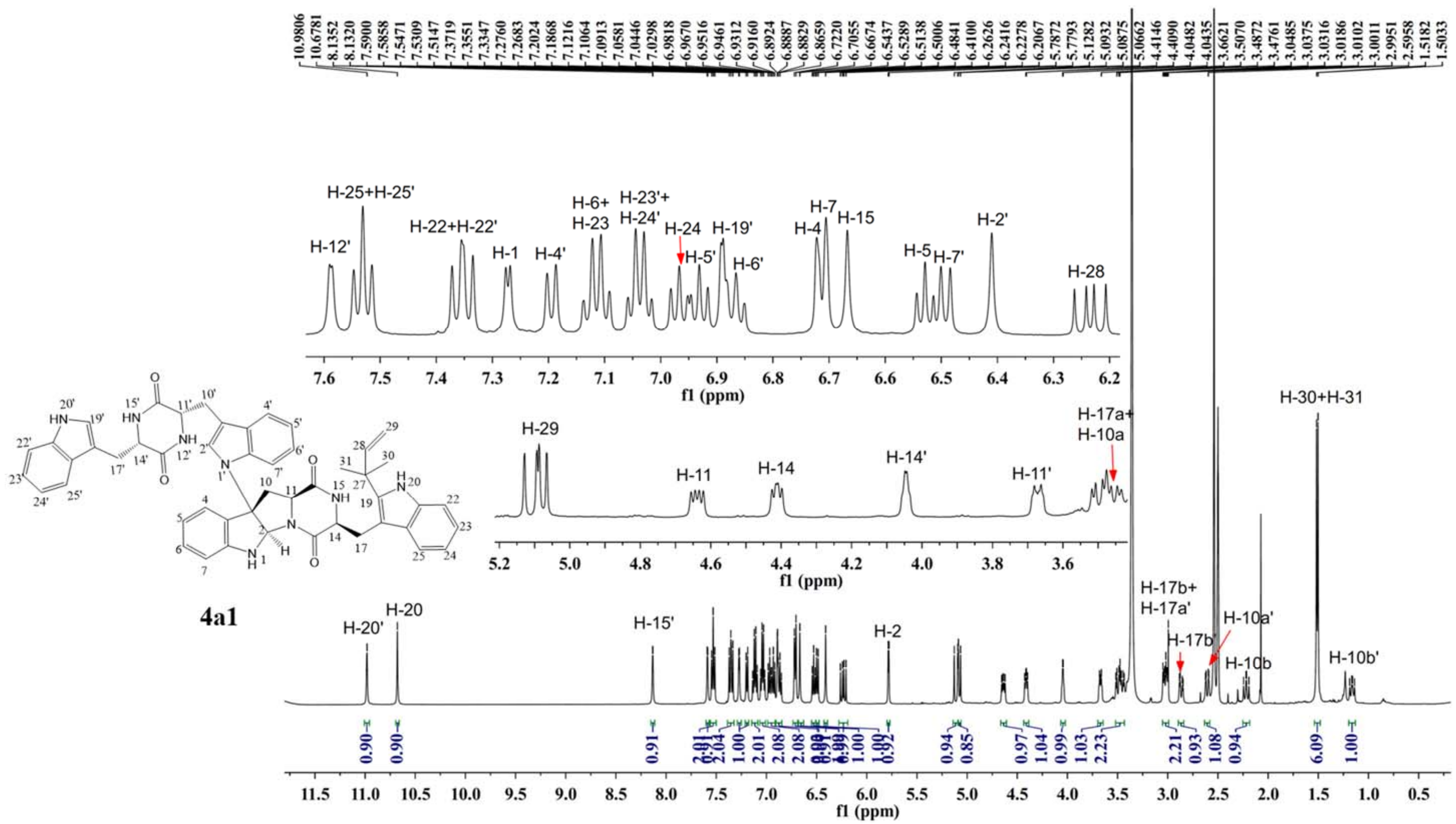

Figure S12. ^{1}H NMR spectrum of **4a1** in DMSO-d_6 (500 MHz).

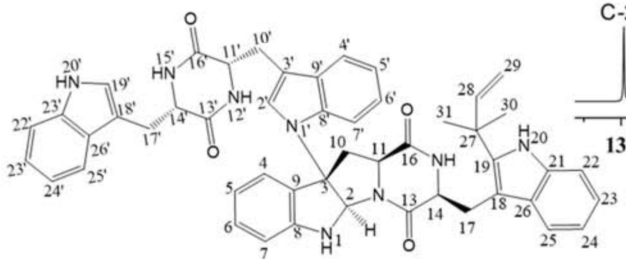

4a1

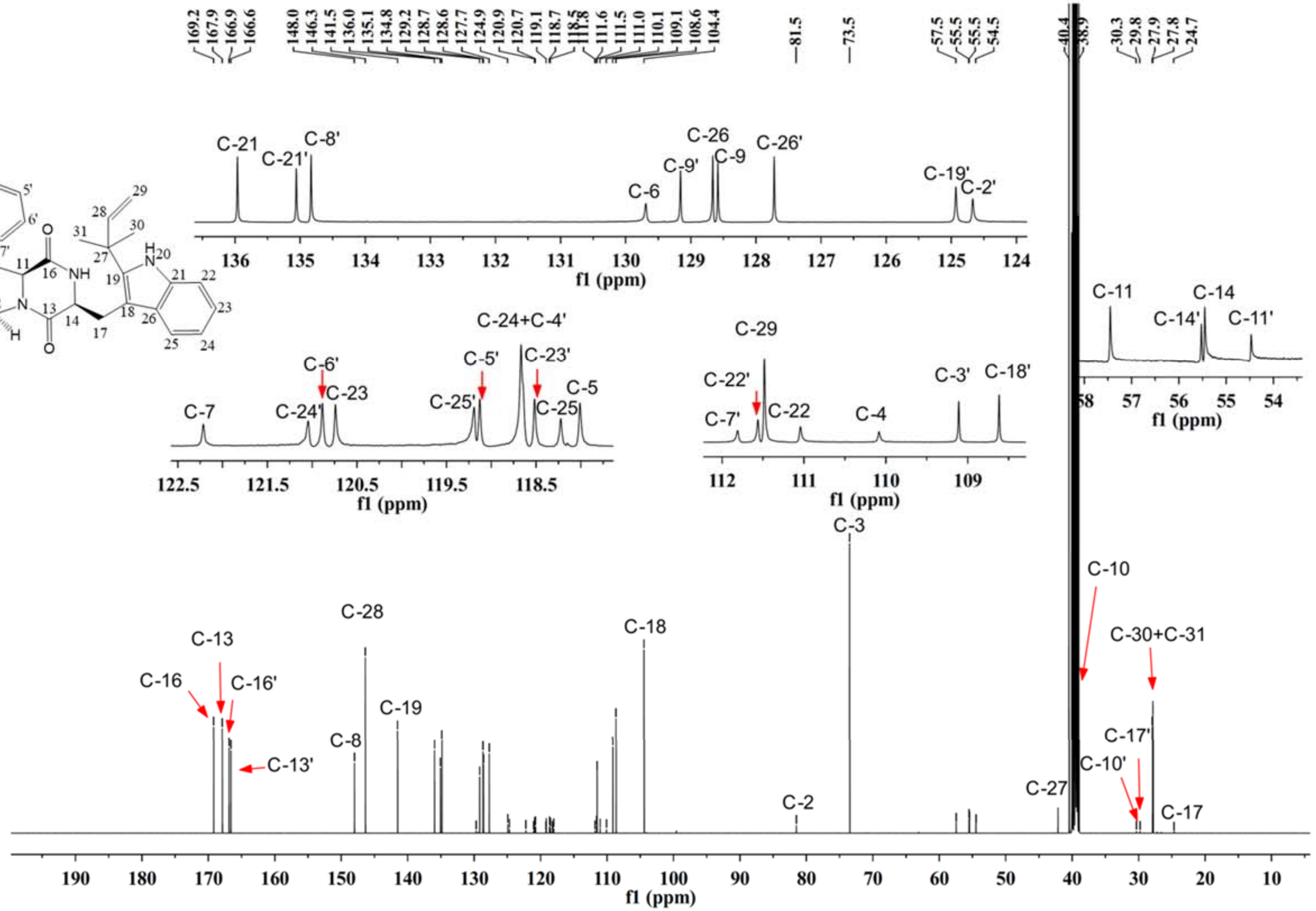

Figure S13. ^{13}C NMR spectrum of **4a1** in DMSO-d_6 (125 MHz).

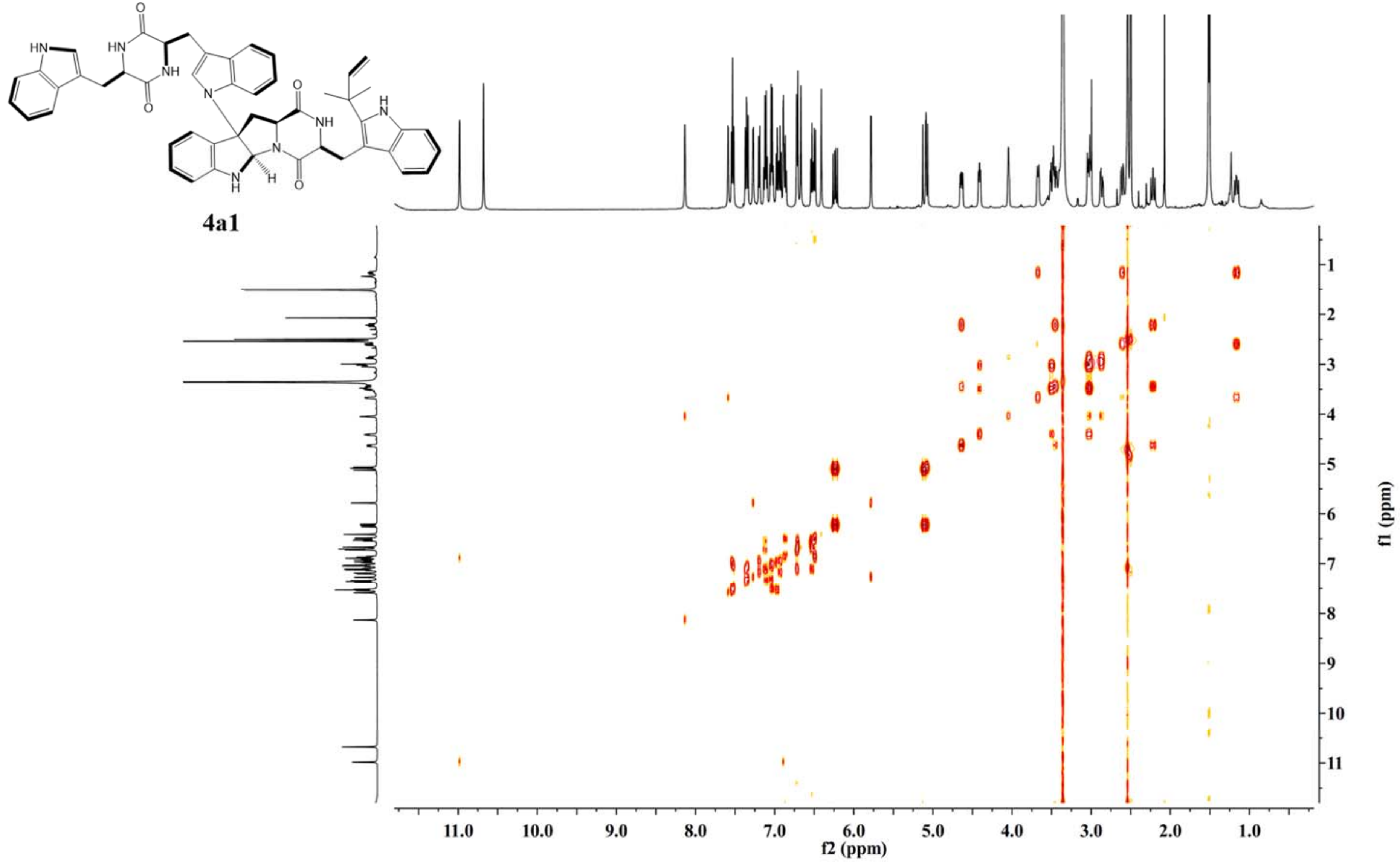

Figure S14. ^{1}H-^{1}H COSY spectrum of **4a1** in DMSO-d_6.

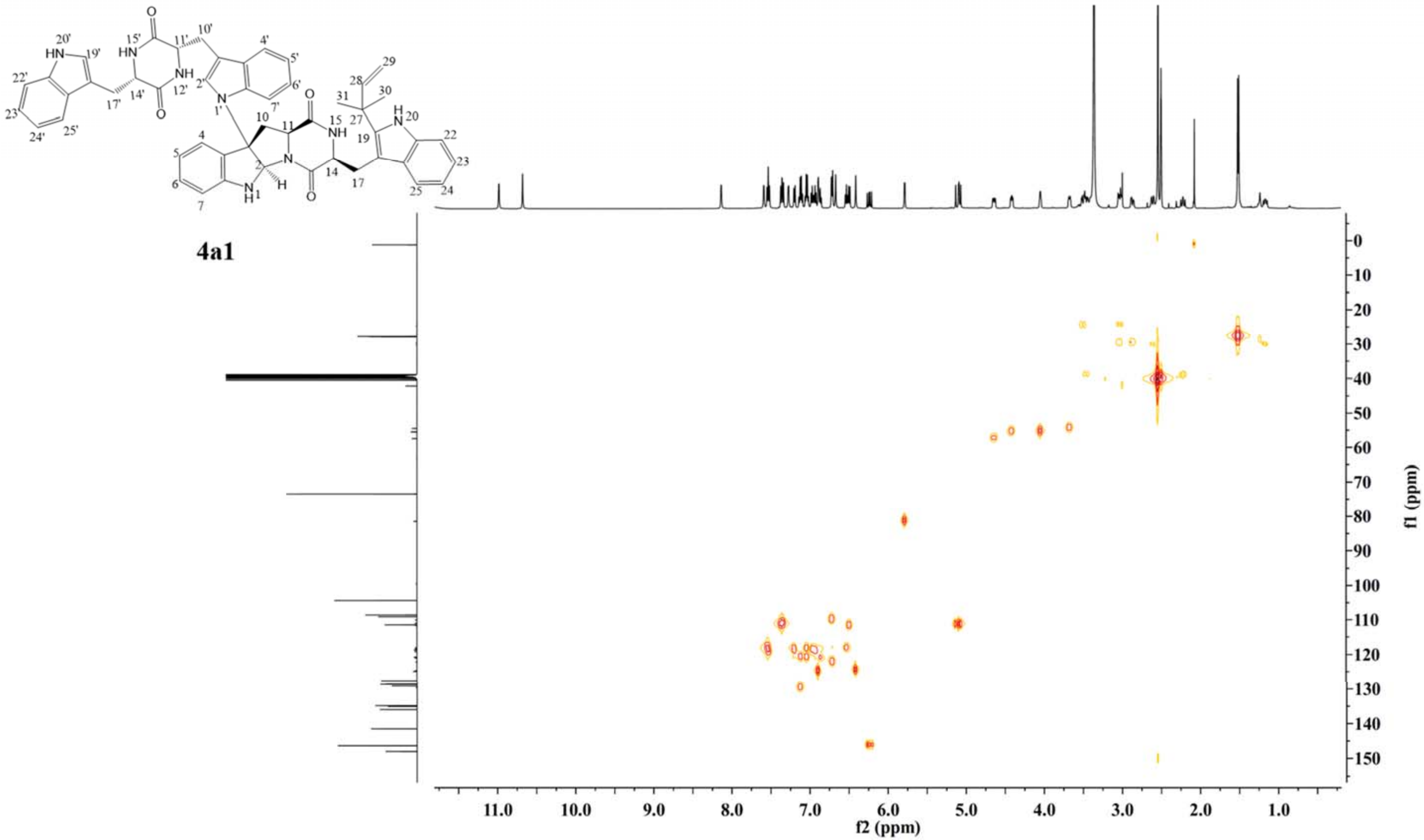

Figure S15. HSQC spectrum of **4a1** in DMSO-d_6.

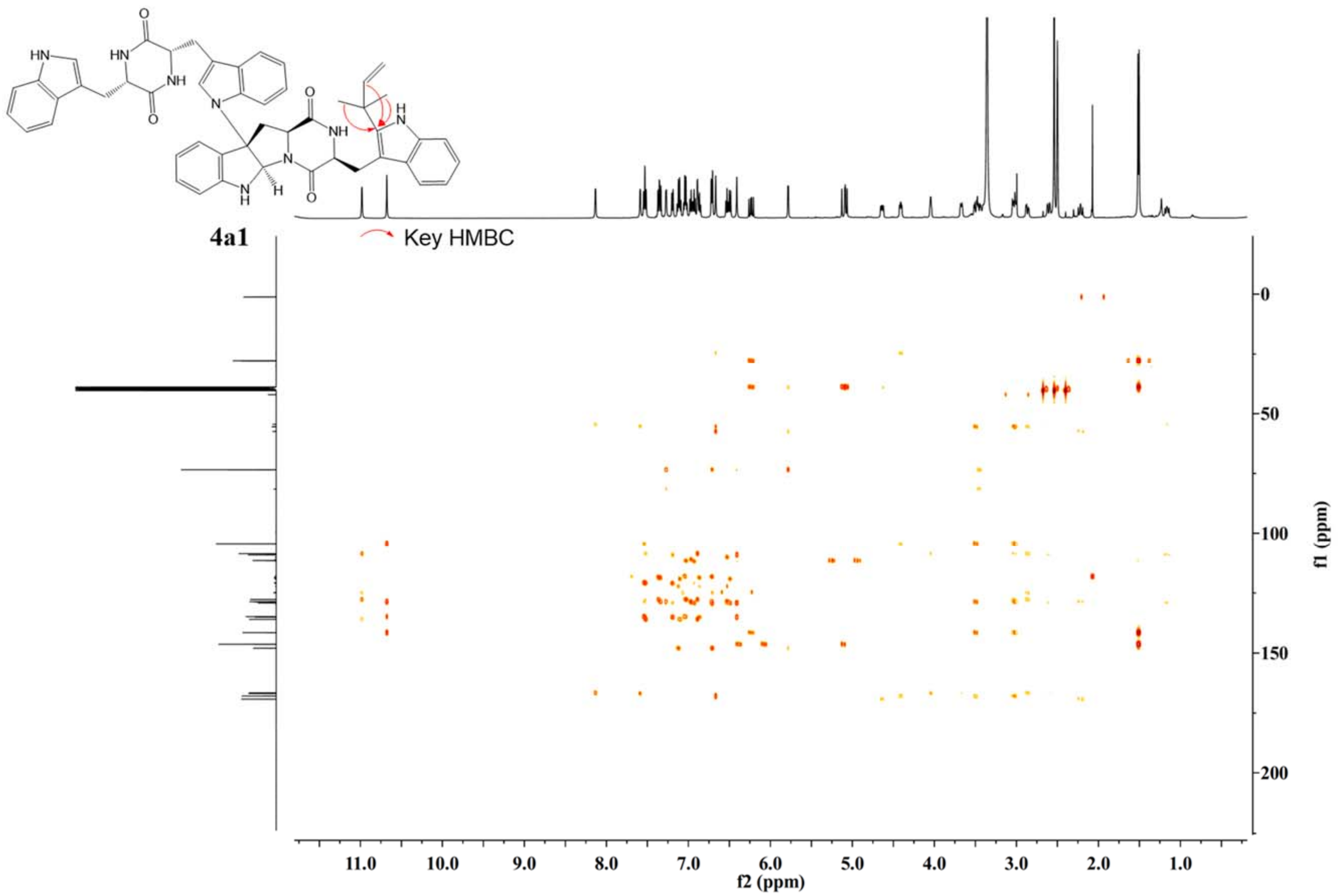

Figure S16. HMBC spectrum of **4a1** in DMSO-d_6.

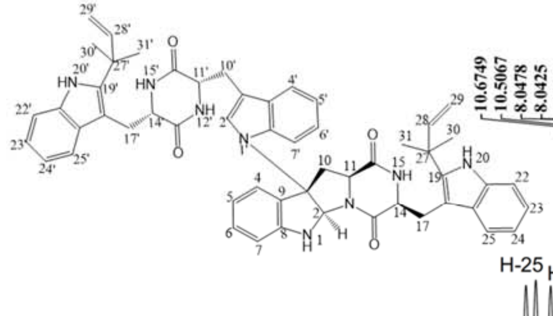

4a2

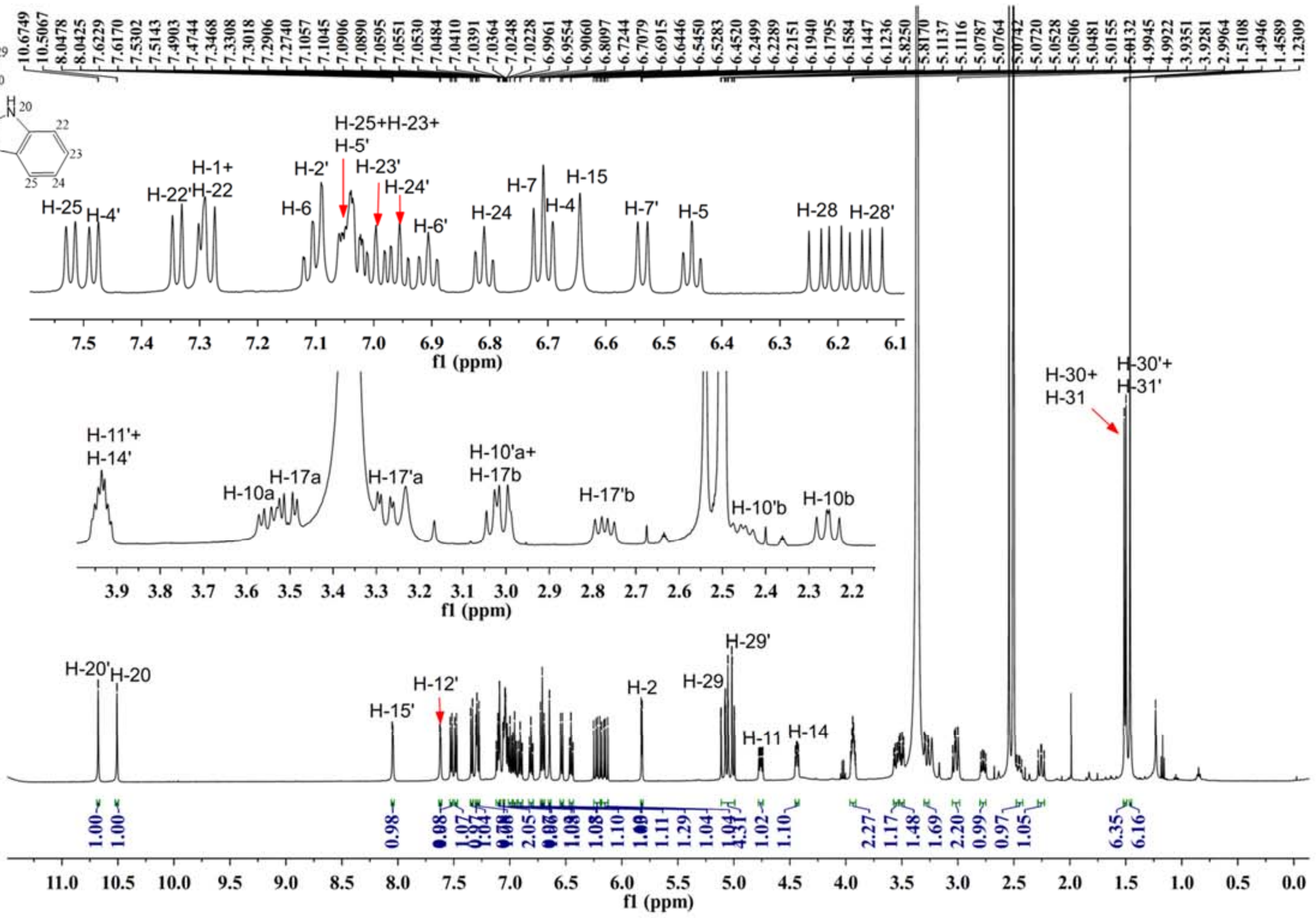

Figure S17. ^{1}H NMR spectrum of **4a2** in DMSO-d_6 (500 MHz).

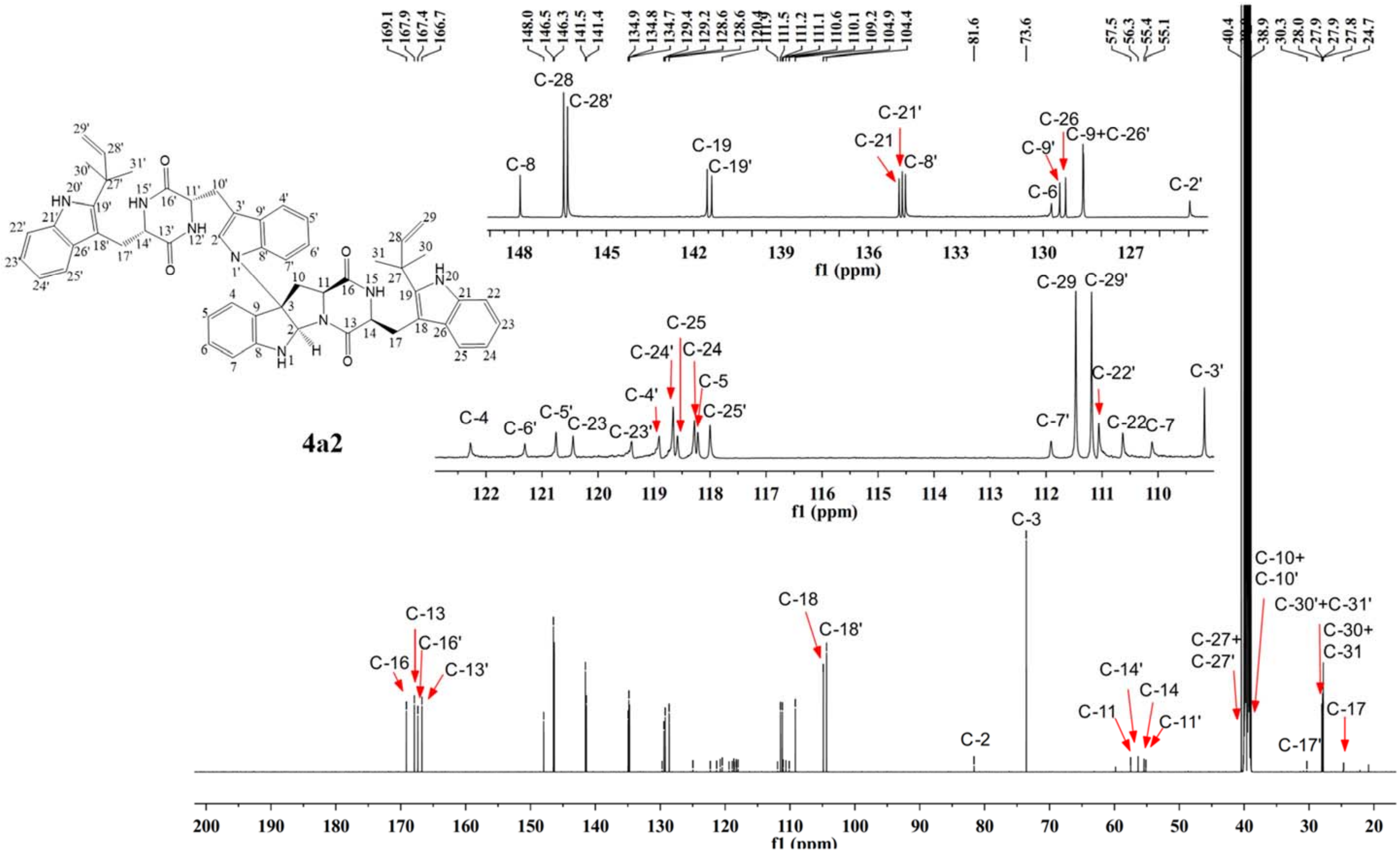

Figure S18. ^{13}C NMR spectrum of **4a2** in DMSO-d_6 (125 MHz).

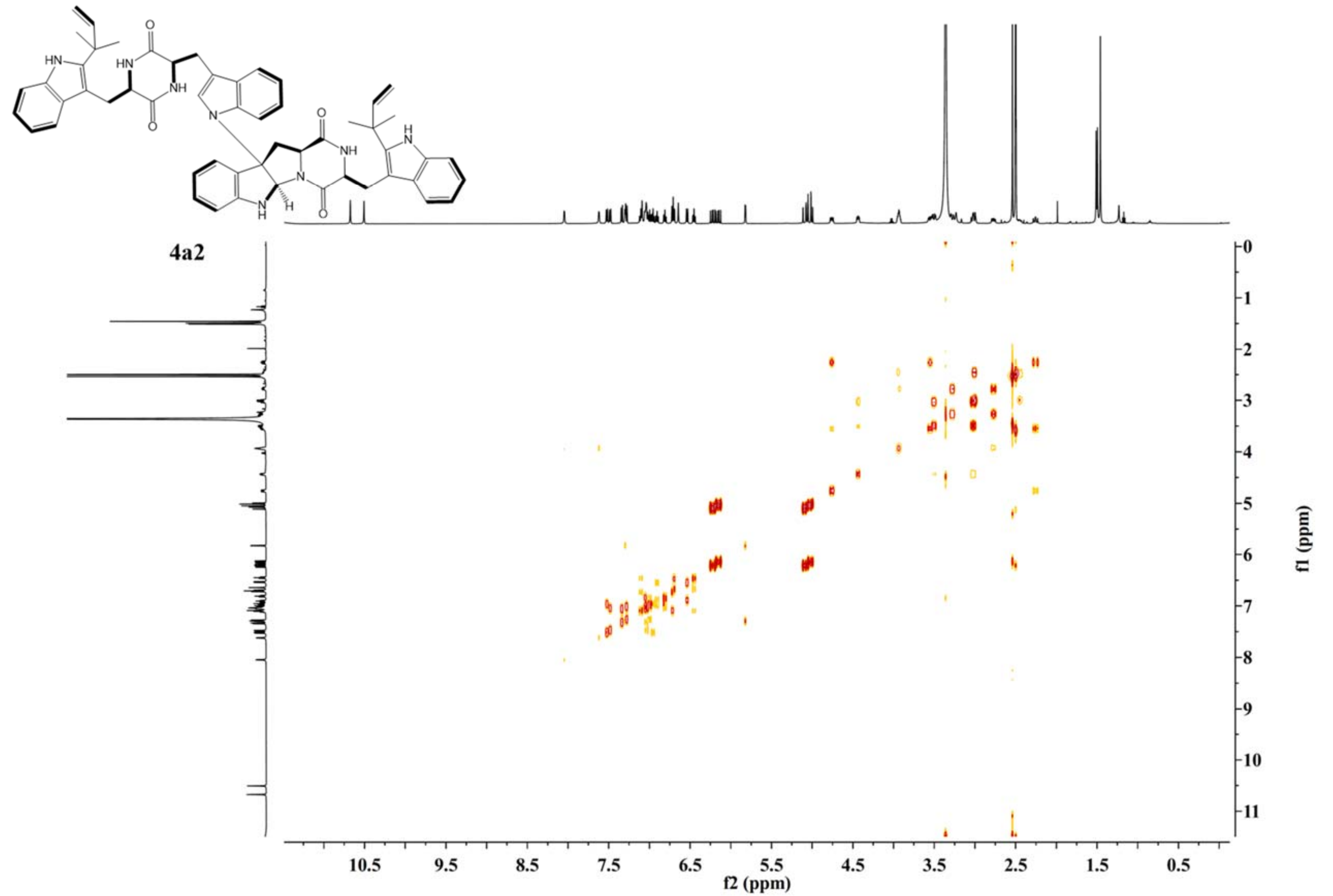

Figure S19. ^{1}H-^{1}H COSY spectrum of **4a2** in DMSO-d_6.

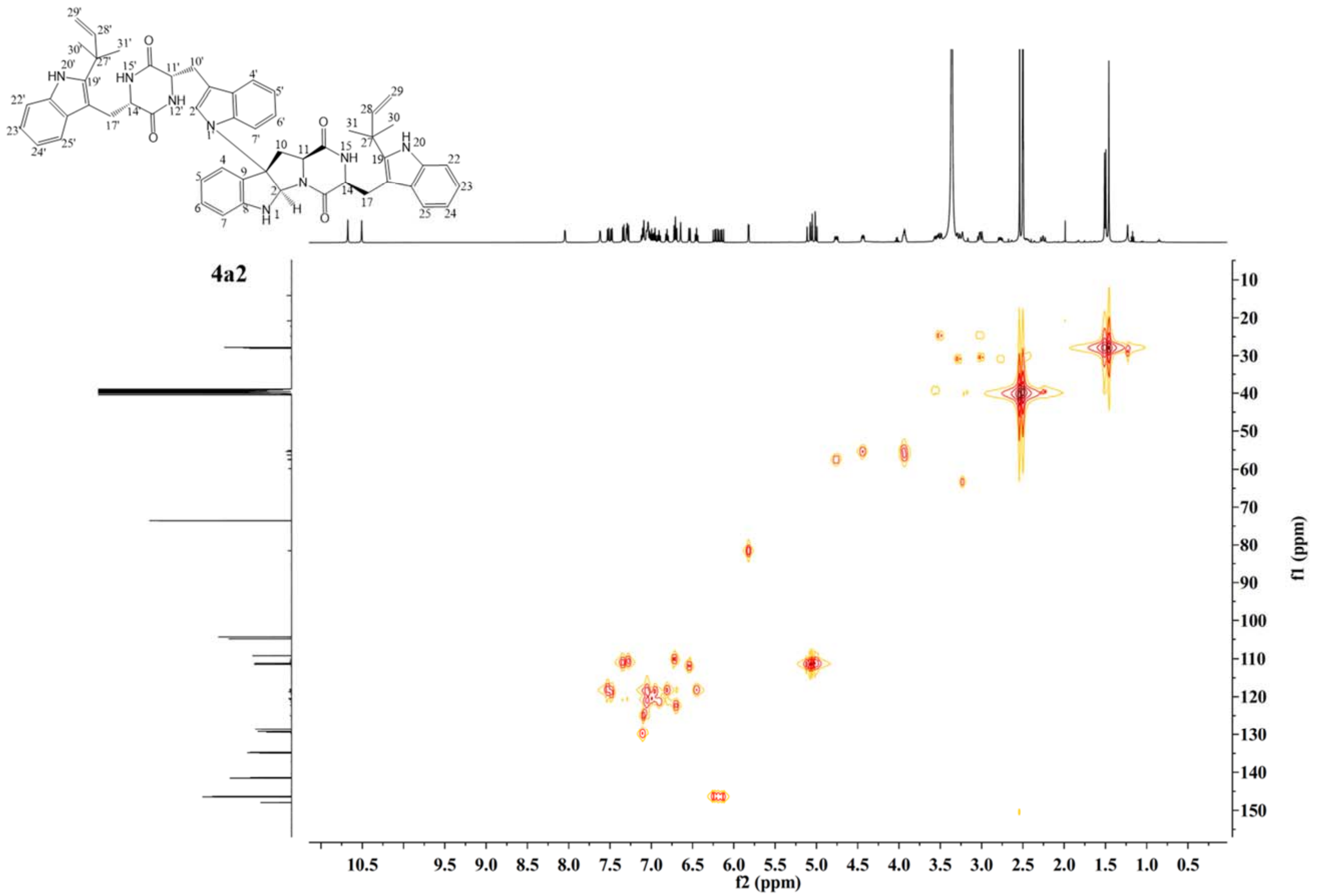

Figure S20. HSQC spectrum of **4a2** in DMSO-d_6.

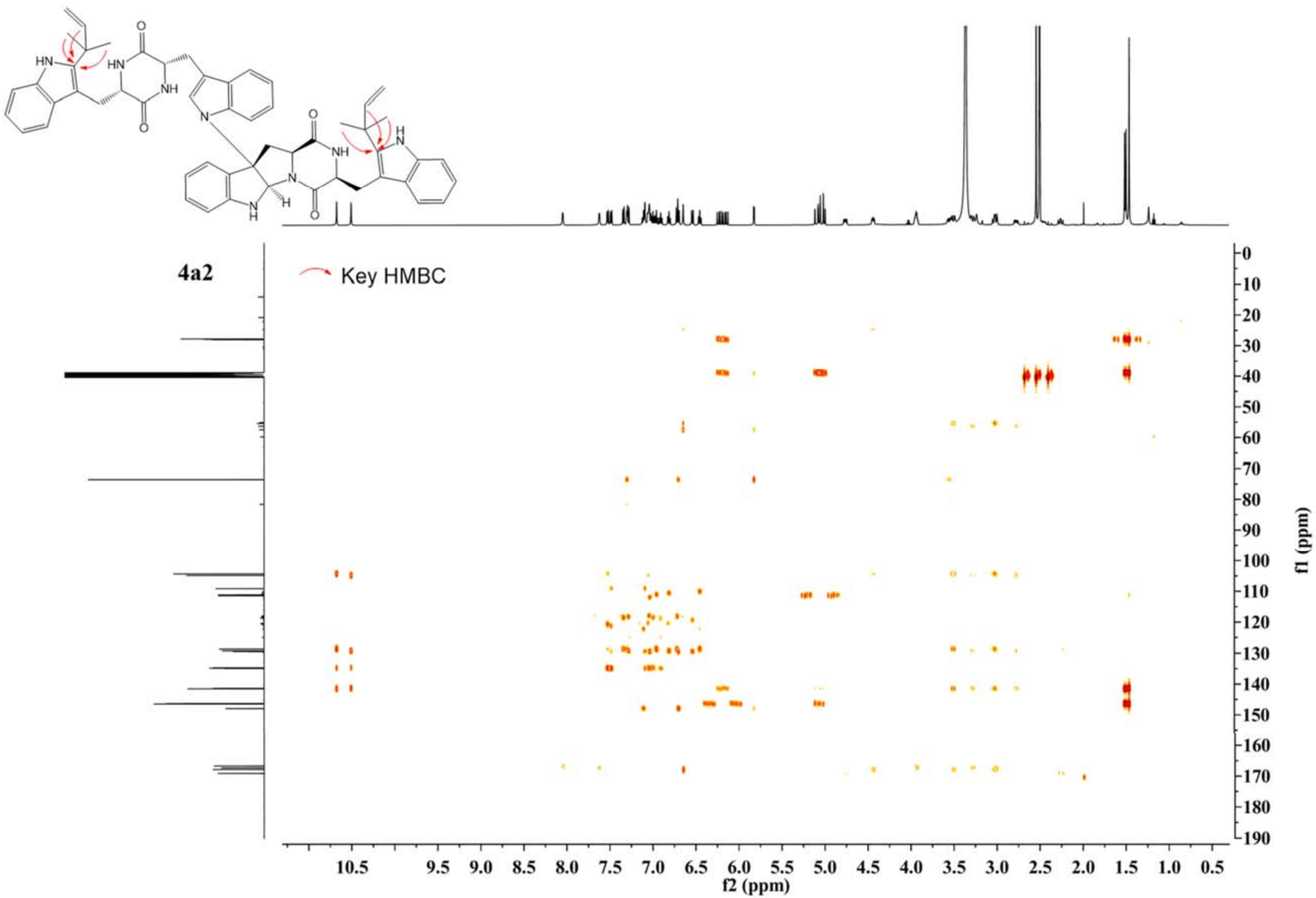

Figure S21. HMBC spectrum of **4a2** in DMSO-d_6.

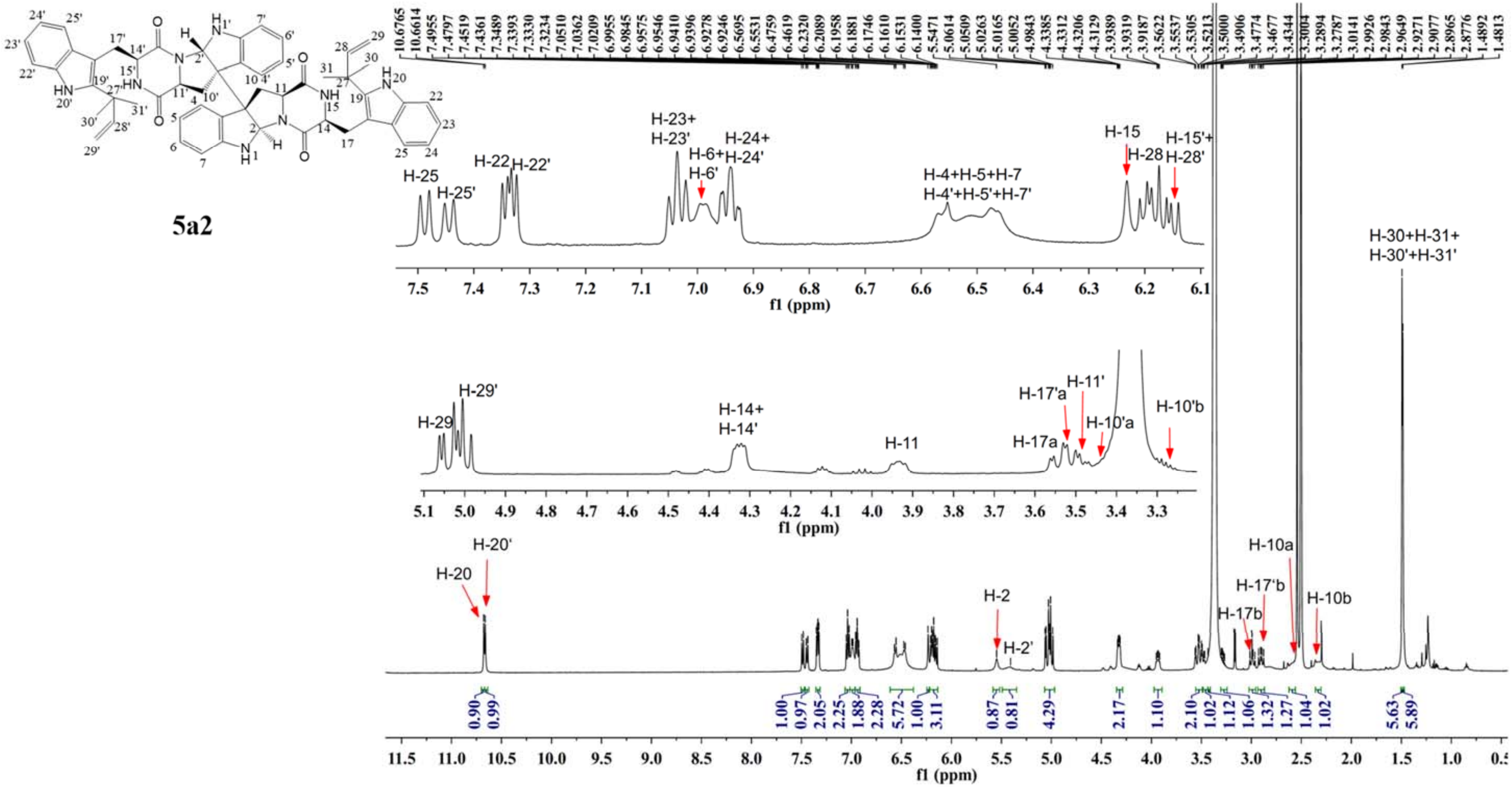

Figure S22. ^{1}H NMR spectrum of **5a2** in DMSO-d_6 (500 MHz).

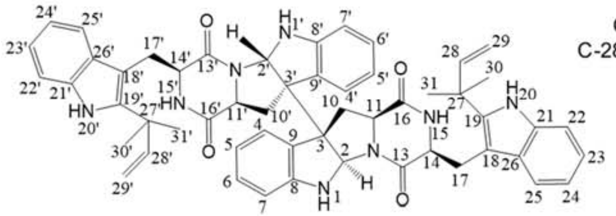

5a2

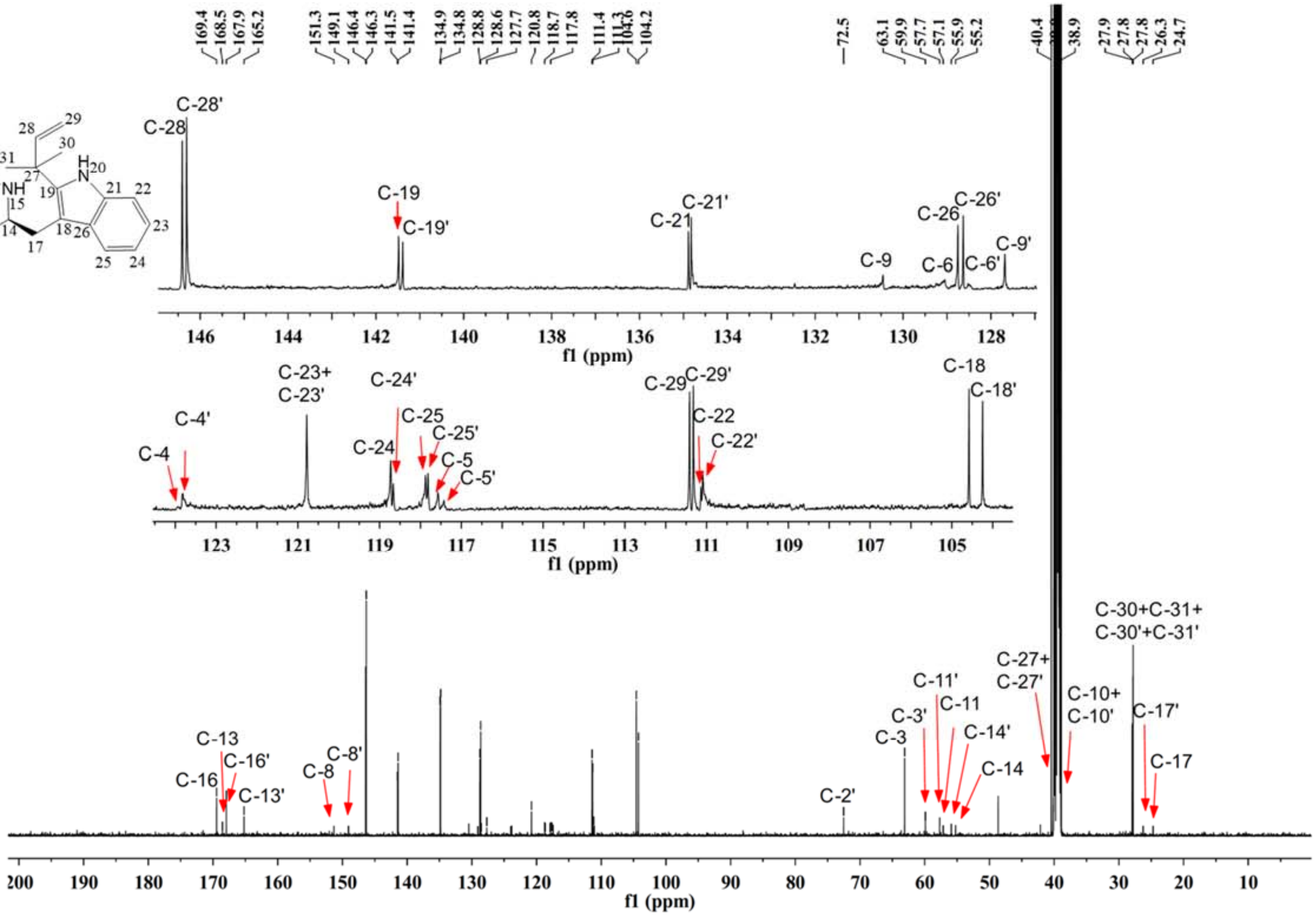

Figure S23. ^{13}C NMR spectrum of **5a2** in DMSO-d_6 (125 MHz).

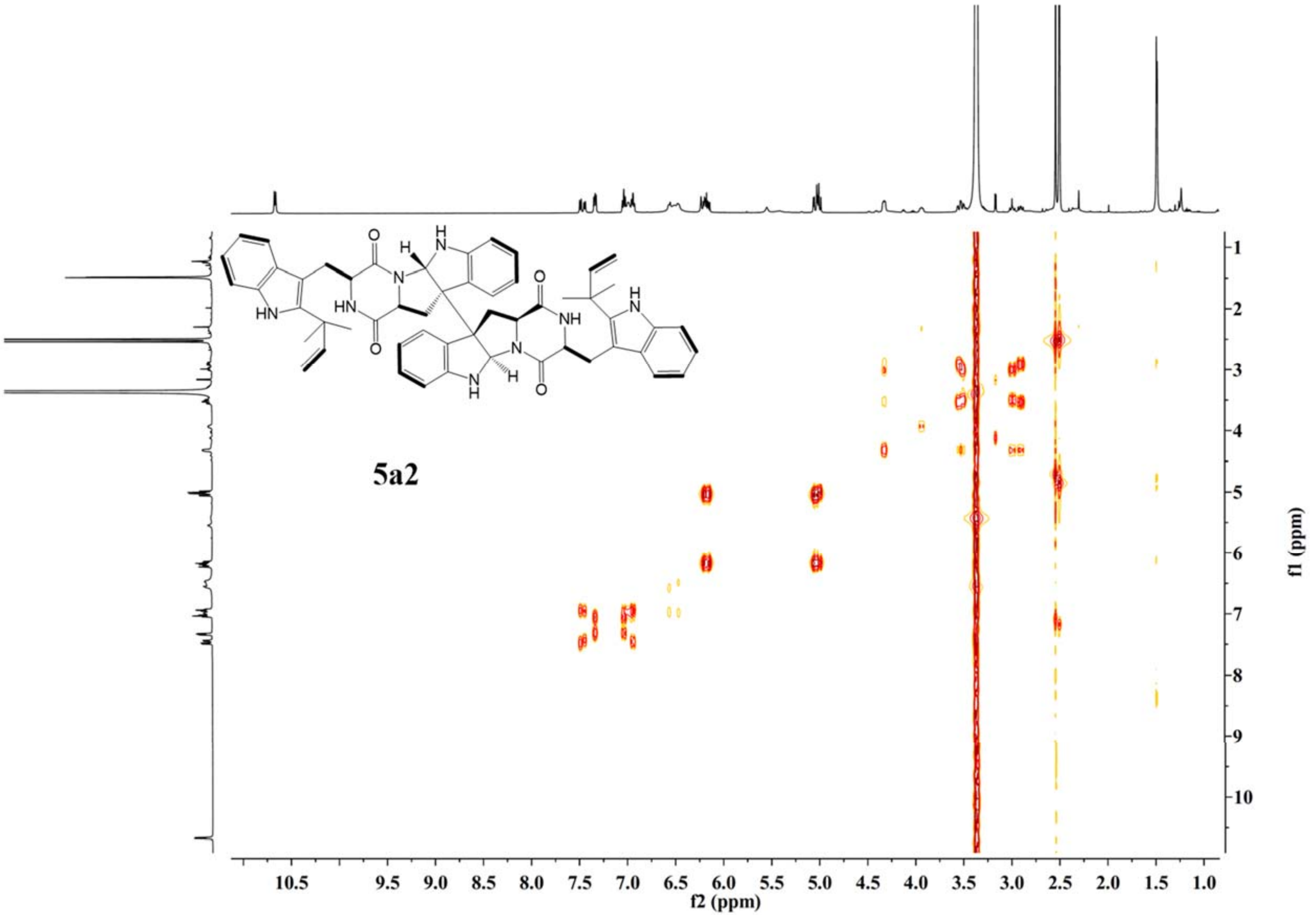

Figure S24. ^{1}H-^{1}H COSY spectrum of **5a2** in DMSO-d_6.

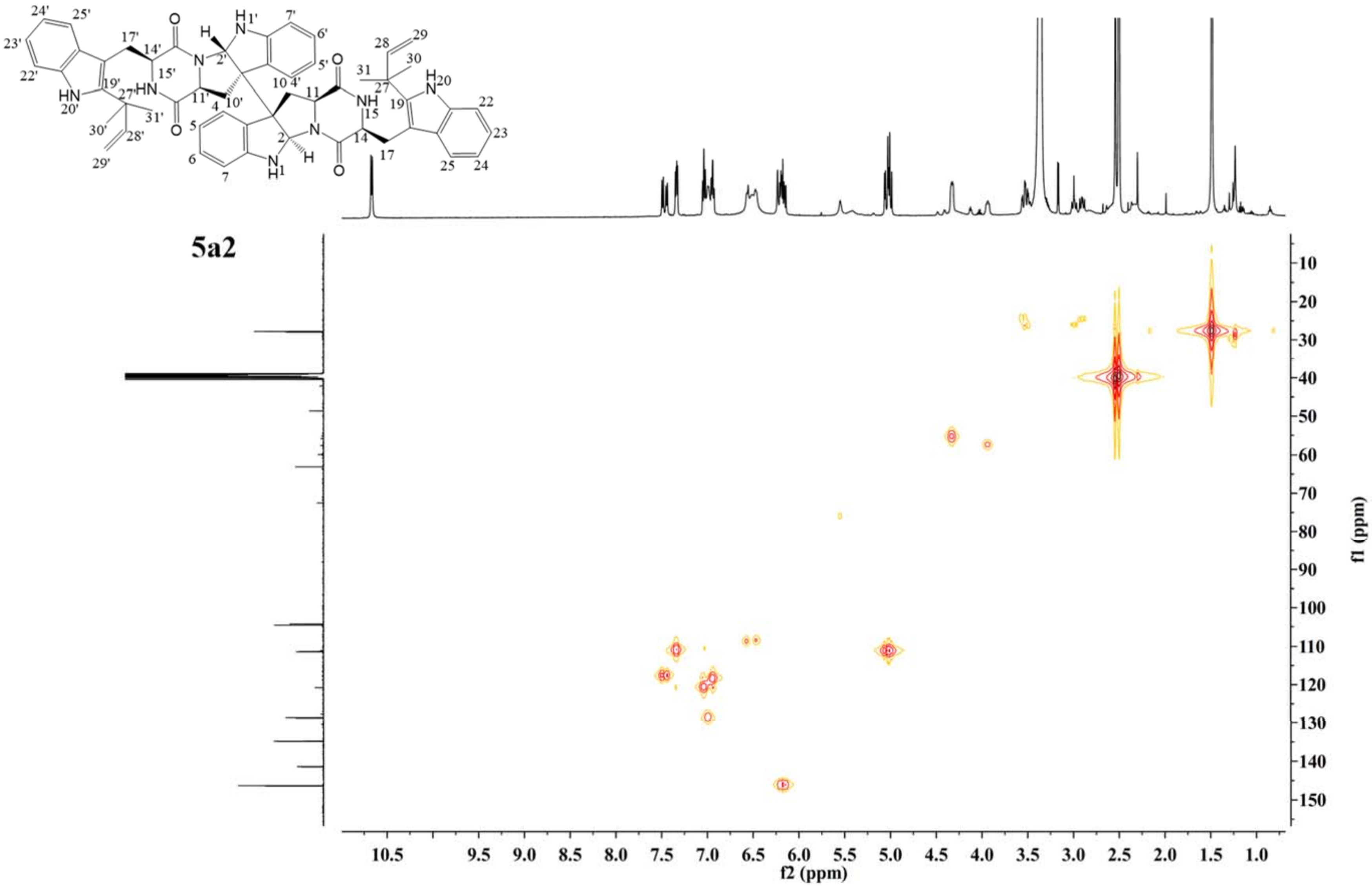

Figure S25. HSQC spectrum of **5a2** in DMSO-d_6.

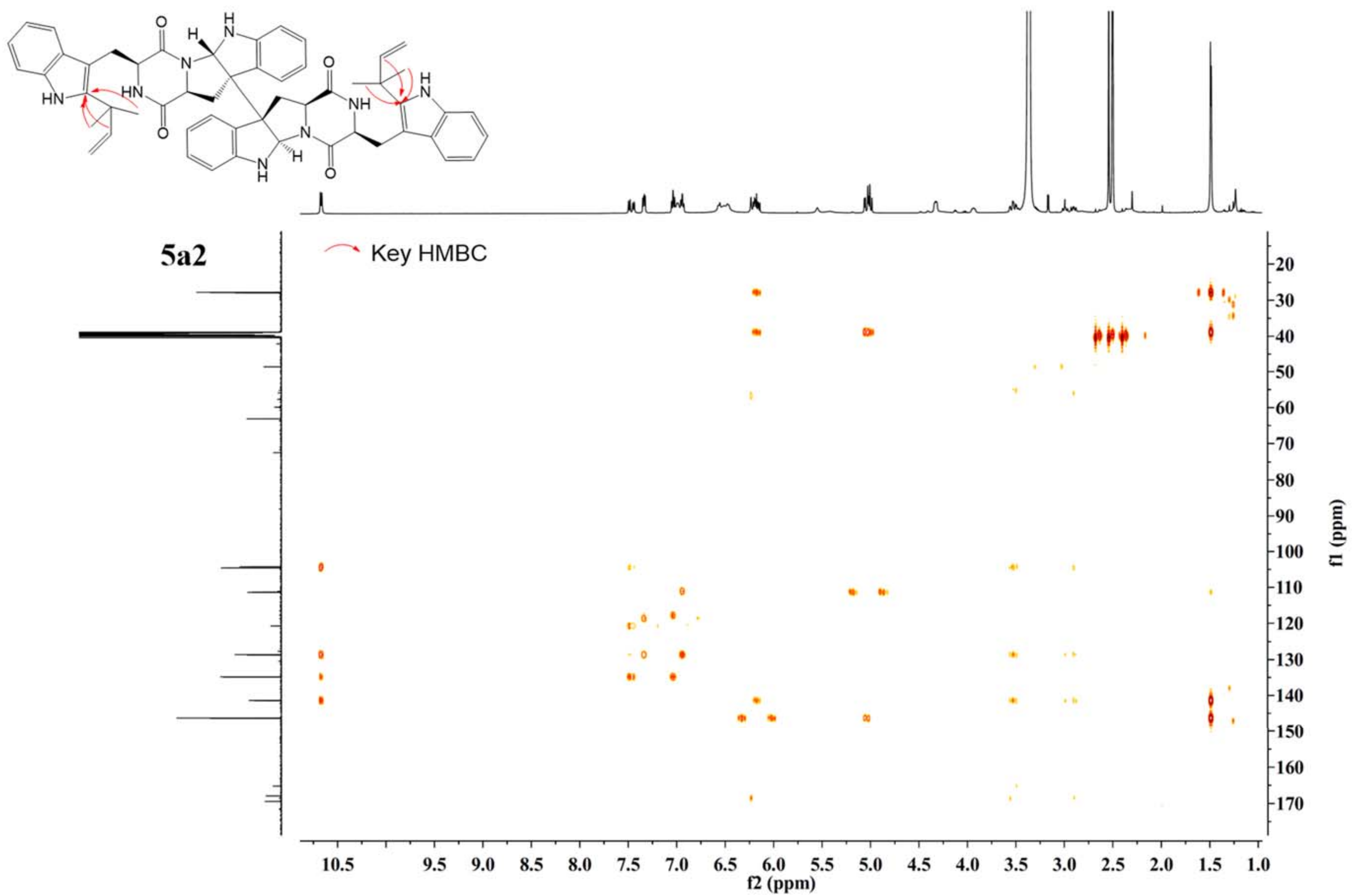

Figure S26. HMBC spectrum of **5a2** in DMSO-d_6.

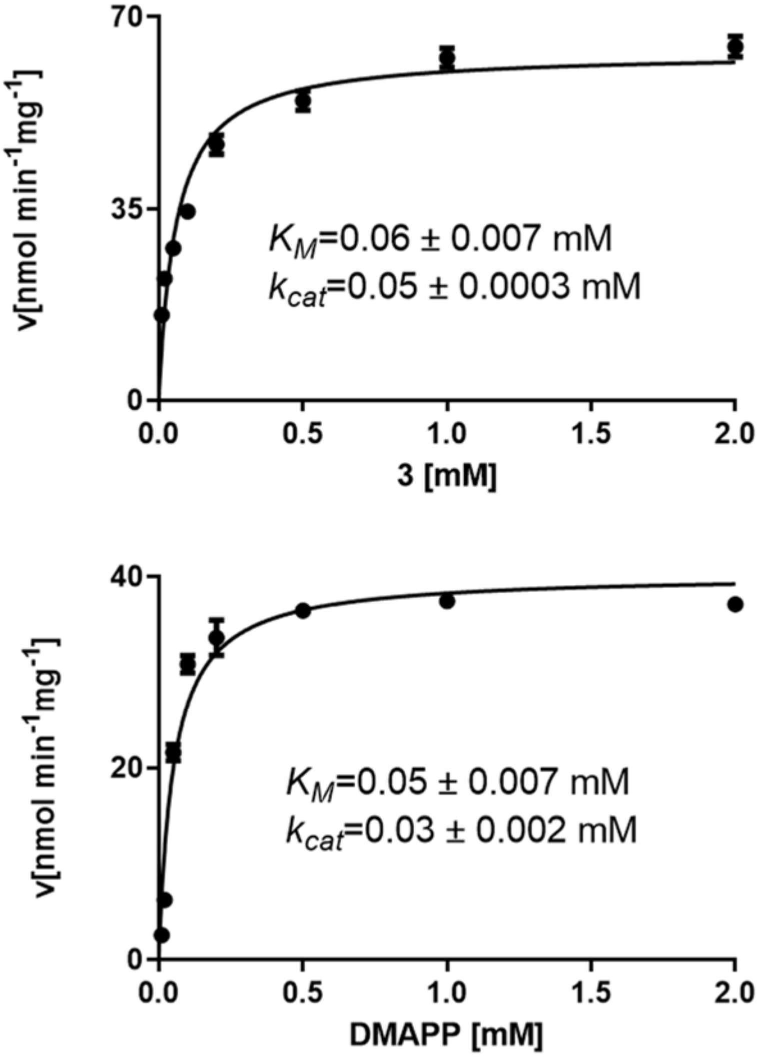

Figure S27. Determination of the kinetic parameters of EchPT1 for **3a2** formation toward **3** and DMAPP. The data were obtained from three independent measurements and the error bars represent the standard errors.

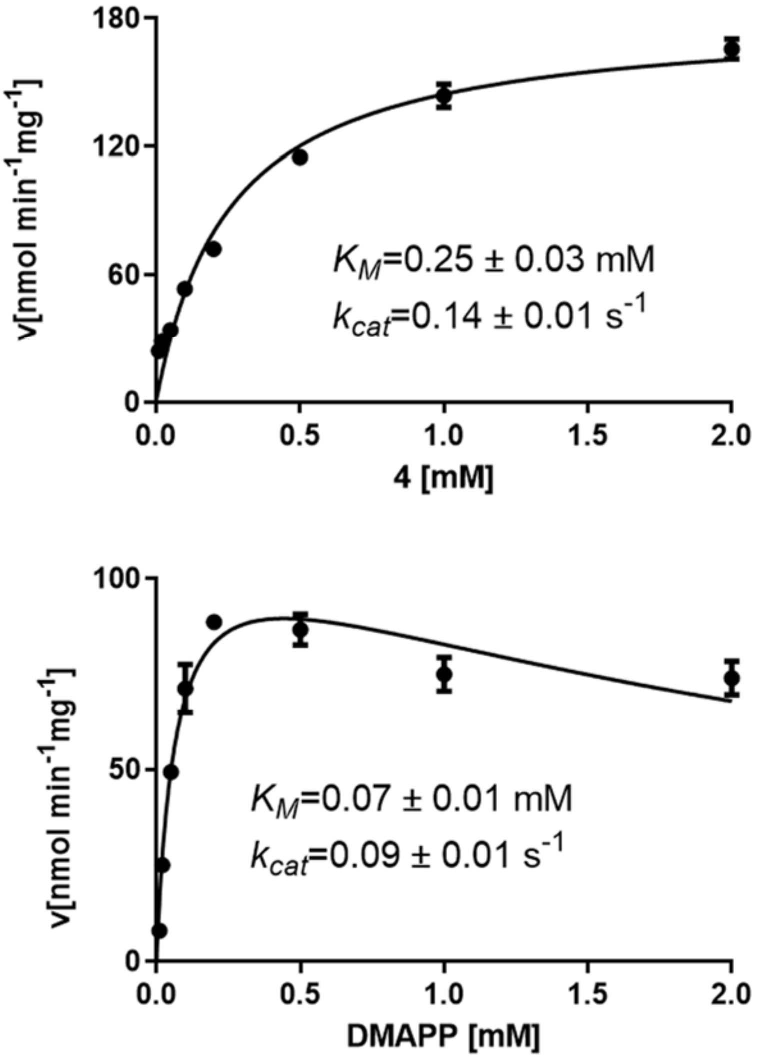

Figure S28. Determination of the kinetic parameters of EchPT1 for **4a1** formation toward **4** and DMAPP. The best curve fit for **4a1** formation toward DMAPP was achieved using a substrate inhibition model in GraphPad Prism 8.01. The data were obtained from three independent measurements and the error bars represent the standard errors.

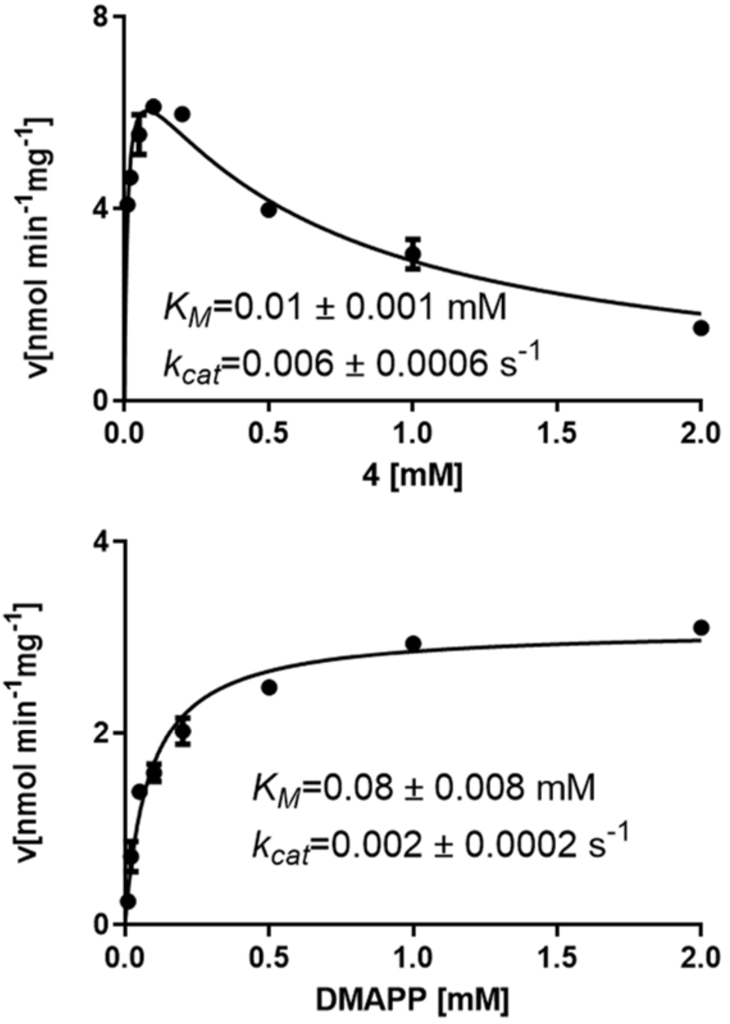

Figure S29. Determination of the kinetic parameters of EchPT1 for **4a2** formation toward **4** and DMAPP. The best curve fit for **4a2** formation toward **4** was achieved using a substrate inhibition model in GraphPad Prism 8.01. The data were obtained from three independent measurements and the error bars represent the standard errors.

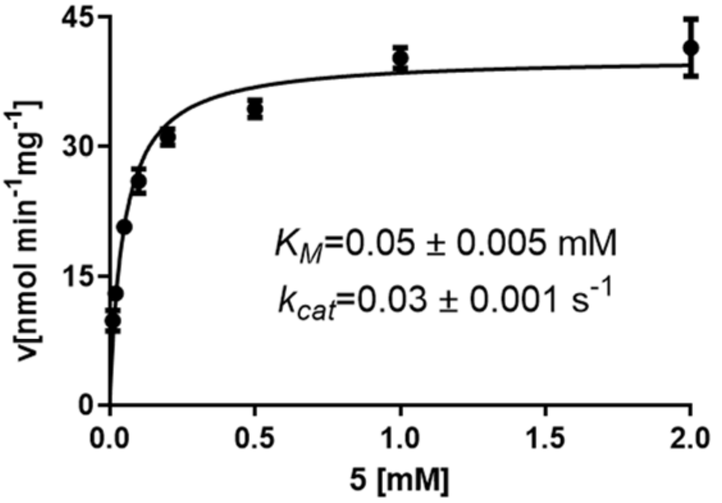

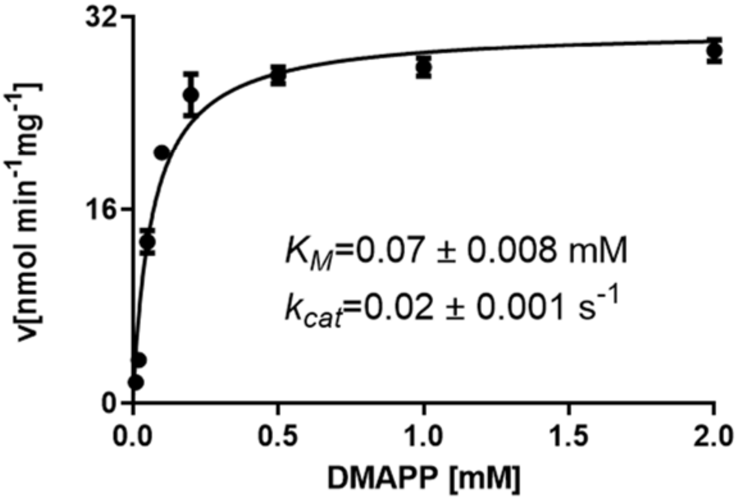

Figure S30. Determination of the kinetic parameters of EchPT1 for **5a2** formation toward **5** and DMAPP. The data were obtained from three independent measurements and the error bars represent the standard errors.
